# Short X···N Halogen Bonds With Hexamethylenetetraamine as the Acceptor

**DOI:** 10.3389/fchem.2021.623595

**Published:** 2021-04-29

**Authors:** Goulielmina Anyfanti, Antonio Bauzá, Lorenzo Gentiluomo, João Rodrigues, Gustavo Portalone, Antonio Frontera, Kari Rissanen, Rakesh Puttreddy

**Affiliations:** ^1^Department of Chemistry, University of Jyvaskyla, Jyvaskyla, Finland; ^2^Centro de Química da Madeira, MMRG, Universidade da Madeira, Funchal, Portugal; ^3^Department of Chemistry, Universitat de les Illes Balears, Palma de Mallorca (Balearus), Spain; ^4^Department of Chemistry, “La Sapienza” University of Rome, Rome, Italy; ^5^Faculty of Engineering and Natural Sciences, Tampere University, Tampere, Finland

**Keywords:** halogen bond, hexamethylenetetraamine, HMTA, N-haloimide, dihalogen, interhalogen

## Abstract

Hexamethylenetetramine (HMTA) and *N*-haloimides form two types of short (imide)X···N and X–X···N (X = Br, I) halogen bonds. Nucleophilic substitution or ligand-exchange reaction on the peripheral X of X–X···N with the chloride of *N*-chlorosuccinimide lead to Cl–X···N halogen-bonded complexes. The 1:1 complexation of HMTA and ICl manifests the shortest I···N halogen bond [2.272(5) Å] yet reported for an HMTA acceptor. Two halogen-bonded organic frameworks are prepared using 1:4 molar ratio of HMTA and *N*-bromosuccinimide, each with a distinct channel shape, one possessing oval and the other square grid. The variations in channel shapes are due to tridentate and tetradentate (imide)Br···N coordination modes of HMTA. Density Functional Theory (DFT) studies are performed to gain insights into (imide)X···N interaction strengths (ΔE_int_). The calculated ΔE_int_ values for (imide)Br···N (−11.2 to −12.5 kcal/mol) are smaller than the values for (imide)I···N (−8.4 to −29.0 kcal/mol). The DFT additivity analysis of (imide)Br···N motifs demonstrates Br···N interaction strength gradually decreasing from 1:1 to 1:3 HMTA:*N*-bromosuccinimide complexes. Exceptionally similar charge density values ρ(r) for N–I covalent bond and I···N non-covalent bond of a (saccharin)N–I···N motif signify the covalent character for I···N halogen bonding.

## Introduction

Halogen bonding, an attractive interaction between the electrophilic region associated with a halogen [X] and a nucleophile [B] forming X···B non-covalent interaction (Desiraju et al., 2013), was recognized by Colin over one and a half centuries ago (Colin, 1814). However, the first X-ray crystal structure evidence of a Br···O halogen bond (XB) in a co-crystal (dioxane)·Br_2_ (Hassel et al., 1954) is considered as a ″turning point″ in subsequent research, development, and application of X···N/O/S motifs in more than one area (Metrangolo et al., 2008; Ho, 2015; Metrangolo and Resnati, 2015). The profound interest in X···N interactions is due to *N*-heteroaromatics widespread structures in nature (Gilday et al., 2015; Cavallo et al., 2016; Lim and Beer, 2018). Crystal engineering studies help promote a better understanding of the X···N(*sp*^2^) X-bonding, and their structures have applications ranging from chemical and optical (Christopherson et al., 2018; Zhuo et al., 2018; Huang et al., 2019; Li et al., 2020) to the preparation of intriguing topologies (Turunen et al., 2017a,b; Vanderkooy et al., 2019). Equally important is the role of NMR (Erdelyi, 2012; Beale et al., 2013; Carlsson et al., 2015) and Density Functional Theory (DFT) (Clark et al., 2007; Politzer et al., 2013, 2017; Riley et al., 2013) studies in efforts to understand the properties of structures in solution.

**Figure 1:**
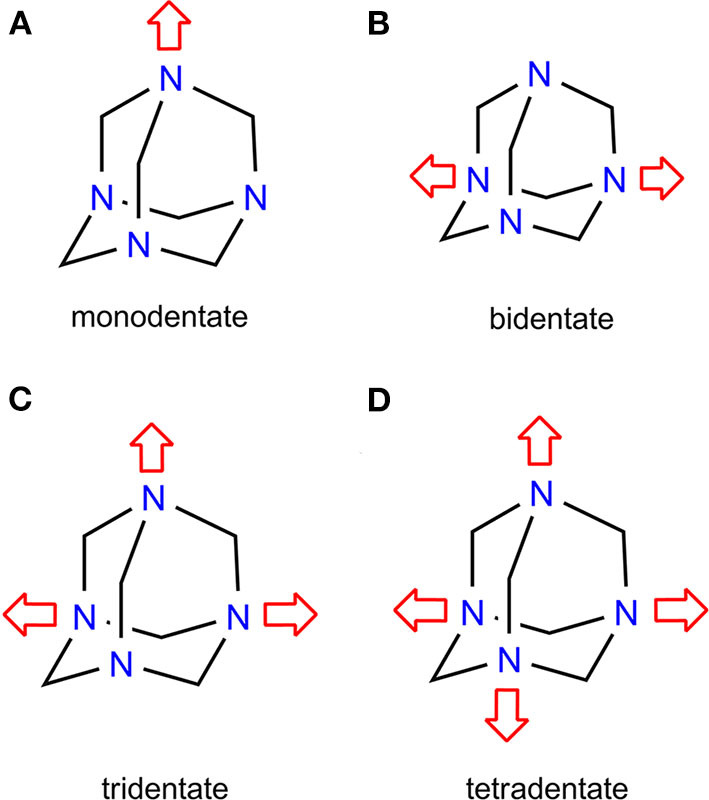
**(A–D)** Overview of HMTA coordination modes.

Unlike X···N(*sp*^2^) interactions, the X···N(*sp*^3^) involving non-aromatic *N*-heterocycles represents a relatively unexplored area. Non-aromatics, such as 1,4-diazabicylcooctane (DABCO) and hexamethylenetetramine (HMTA), have become a critical design trait in supramolecular chemistry, and have inspired scholars to devise molecular rotors (Catalano et al., 2015) and organic frameworks (XBOFs) (Raatikainen and Rissanen, 2012). HMTA produces an array of structural diversity due to its peculiar diamondoid-like skeleton and polydentate coordination nature. A Cambridge Structural Database (CSD, 2020) search for HMTA functioning as an XB acceptor revealed 27 hits, of which 2, 12, 4, and 8 hits correspond to mono- (Eia et al., 1956; Pritzkow, 1975a), bi- (Eia et al., 1956; Dahl et al., 1971; Pritzkow, 1975b; Walsh et al., 2001; Raatikainen and Rissanen, 2011; Syssa-Magalé et al., 2014; Gonzalez et al., 2017; Szell et al., 2019), tri- (Hassel et al., 1954; Eia et al., 1956; Dahl and Hassel, 1965), and tetradentate (Pritzkow, 1974; Raatikainen and Rissanen, 2011, 2012) coordination modes (Figure 1). Bidentate HMTA is frequently encountered in halogen- and hydrogen-bonded complexes. The control over the bidentate mode depends on the HMTA itself while the tetradentate relies heavily on the donor, solvents, hydrogen bonds (HBs), and packing forces (Lemmerer, 2011). Crystallization experiments involving HMTA and dihalogens, e.g., iodine, often generate an acidic solvent medium yielding undesired HB complexes of the sort [HMTA-H]^+^·${\text{I}}_{\text{n}}^{-}$ rather than desired halogen-bonded structures [HMTA]·[I_2_]_n_ (Tebbe and Nagel, 1995b). The protonation ability of HMTA is linked to the *sp*^3^ character of nitrogen. Many other fundamental structural features of HMTA are unknown, and their synthetic and structural rationale could provide useful groundwork in self-assembly design and even pave the way to the rational design of materials.

**Figure 2:**
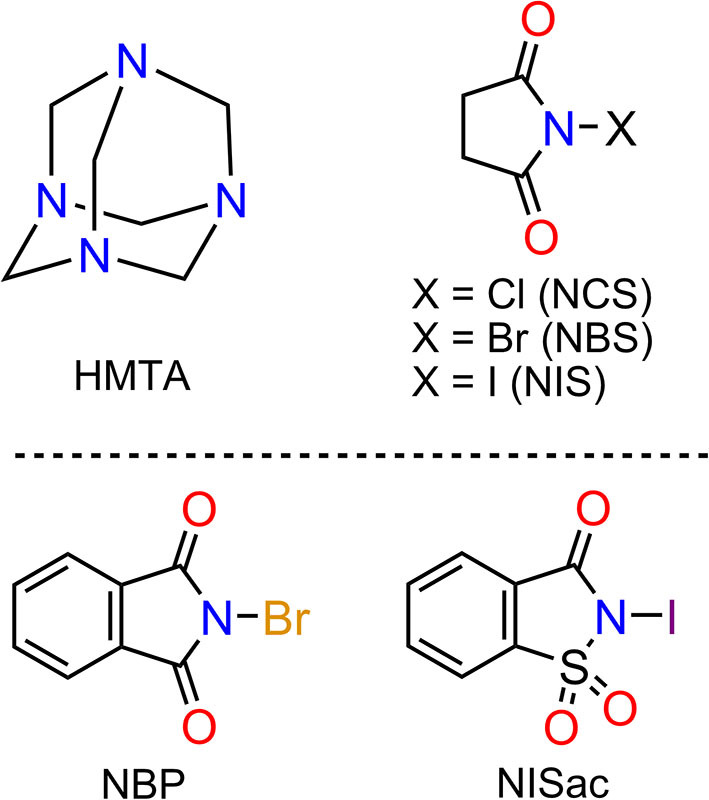
List of components: Hexamethylenetetraamine (HMTA), N-chlorosuccinimide (NCS), N-bromosuccinimide (NBS), N-iodosuccinimide (NIS), N-bromophthalimide (NBP), and N-iodosaccharin (NISac).

Our previous contributions in the field of HMTA halogen bonding include (i) the comparison of Br···N bond distances in bidentate complexes [HMTA]·[N-bromosuccinimide]_2_ and [DABCO]·[N-bromosuccinimide]_2_ (Raatikainen and Rissanen, 2011)_._ The Br···N distances of [HMTA]·[NBS]_2_ [2.414(3) and 2.432(3) Å] are longer than in [DABCO]·[NBS]_2_ [2.347(2) and 2.364(2) Å] due to steric and competitive HB interactions in the former structure. (ii) Solvent as the only varying parameter, six different [HMTA]·[N-iodosuccinimide]_4_ XBOFs have been characterized using X-ray diffraction analysis (Raatikainen and Rissanen, 2012). In each of the six XBOFs, one HMTA and four NIS molecules form short I···N XBs [2.486(3) to 2.586(3) Å]. In the present study, the scope of the (imide)X···N(HMTA) motif is expanded using components listed in Figure 2. Our aim is to (i) gain new insights into the coordination modes of HMTA in [HMTA]·[N-haloimide]_n_ complexes, (ii) find the ″ideal″ HMTA:*N-*haloimide partner to access open-framework XBOF structures, and (iii) evaluate and compare the X···N interaction strengths.

## Results and Discussions

### Synthesis

**Scheme 1:**
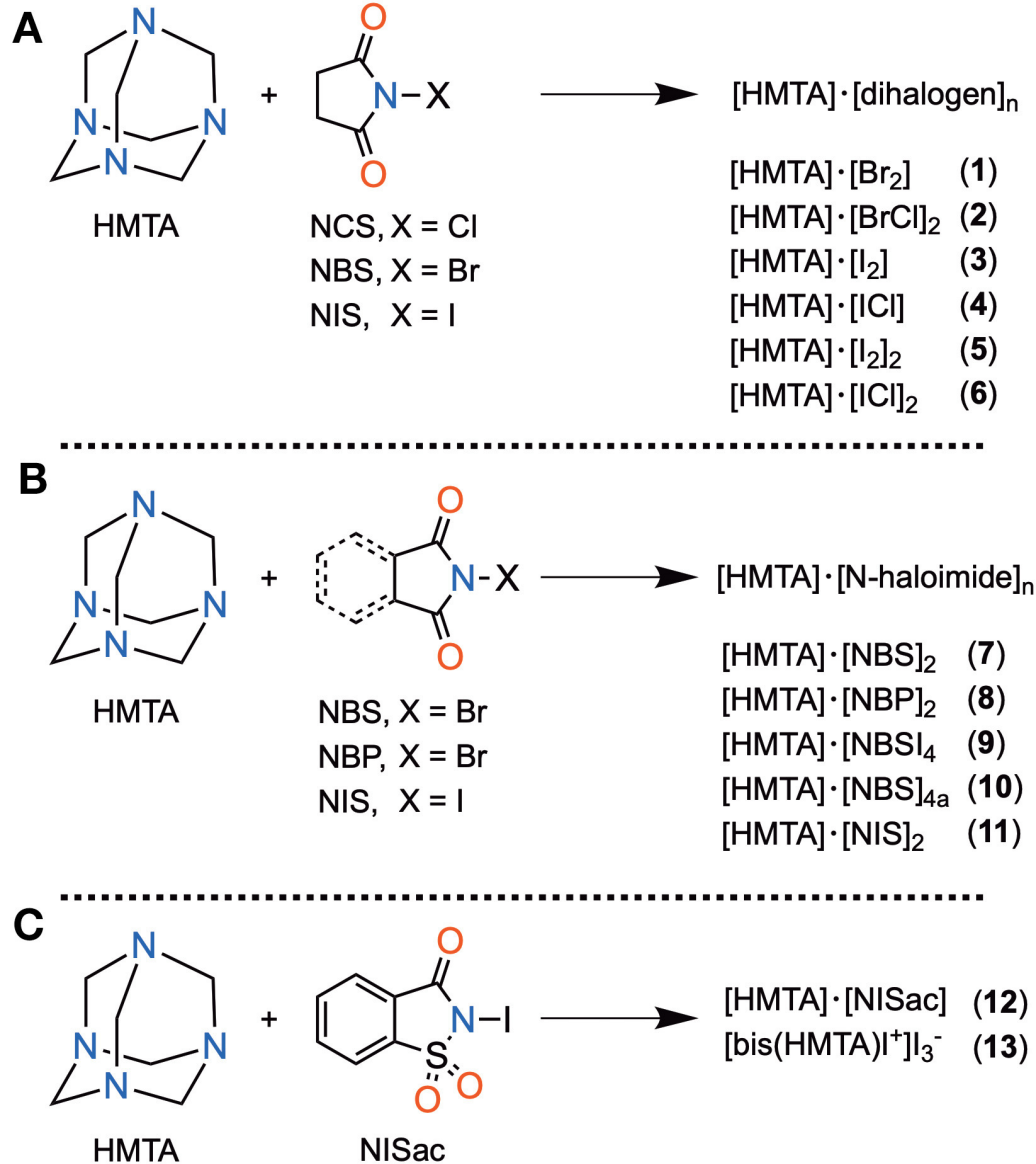
**(A–C)** List of halogen-bonded complexes.

HMTA and five different N-haloimides, namely N-chlorosuccinimide (NCS), N-bromosuccinimide (NBS), N-iodosuccinimide (NCS), N-bromophthalimide (NBP), and N-iodosaccharin (NISac), were used to prepare 13 halogen-bonded complexes **1**–**13** of composition types [HMTA]·[dihalogen]_n_ and [HMTA]·[N-haloimide]_n_ (Scheme 1). [HMTA]·[dihalogen]_n_ is of two types: complexes **1**, **3**, and **5**, which contain homo-halogen X–X···N (X = Br, I) motifs, and complexes **2**, **4**, and **6** comprising hetero-halogen Y–X···N (Y = Cl, X = Br, I) motifs. The Br_2_ source in **1** and I_2_ in **3** and **5** are consequences of N–X bond cleavage reactions of NBS and NIS, respectively (Filler, 1963). The precedented yet unique formation route of X–X···N halogen bonds inspired us to prepare **2**, **4**, and **6**. The Cl–Br···N of **2** and Cl–I···N of **4** and **6** were obtained by using trios HMTA, NCS, and NBS, and HMTA, NCS, and NIS, respectively. A two-step sequential ligand-exchange reaction can be attributed to the formation of Cl–Br/I···N motifs. For example, in the synthesis of **2**, step one involves mixing HMTA and NBS. During this step, the initially formed (CO)_2_N–Br···N gradually converts to Br–Br···N motif by N–Br bond cleavage reaction followed by the exchange of (CO)_2_N and Br anions. In the second step, NCS, a chloride anion source, was added to replace the terminal bromide anion to give **2**.

Single-crystals of **7**–**13** were obtained by slow evaporation of the corresponding HMTA and N-haloimide solutions (for more details, see SI). The 1:4 HMTA:NBS and 1:4 HMTA:NBP molar ratio reactions only gave the corresponding bidentate complexes **7** and **8** as the main products for structural analysis. Single-crystals isolated from different 1:4 HMTA:NBS experiments carried out by using various solvents also revealed the bidentate coordination for HMTA (for more details, see SI). Using a 1:4 HMTA:NBS molar ratio, complexes **9** and **10** were obtained employing different crystallization techniques. Complex **9**, which contains a tridentate Br···N HMTA, was obtained using solvent-assisted grinding followed by solution crystallization, while **10**, determined to be tetradentate Br···N HMTA, was crystallized by using the layering technique. Under the crystallization method of **9**, the other HMTA-imide combinations produced crystals of either HMTA or corresponding imide. Even **9** is not reproducible and yields crystals of succinimide and bidentate complex **7**. The lack of reproducibility is due to the influence of several uncontrolled factors in the crystallization process, such as N–X bond cleavage reactions and complex hydrogen bonding patterns.

Experiments conducted using 1:4 molar ratio of HMTA and NIS in different solvents all exclusively produced crystals of **11**. A 1:2 HMTA:NISac molar ratio gives a monodentate complex **12**. However, treating HMTA with an excess of NISac (12.5 eq) leads to the formation of unknown quantities of iodine-oriented ions consequently resulting in an iodonium complex [bis(HMTA)I]^+^${\text{I}}_{3}^{-}$ **13**. A related method to prepare [bis(HMTA)I]^+^${\text{I}}_{3}^{-}$ in the presence of concentrated iodine ethanol solution previously reported by Bowmaker et al. and Pritzkow (Bowmaker and Hannan, 1971; Pritzkow, 1975a) supports our hypothesis. Our attempts to crystallize the 1:4 ratio complexes of HMTA and iodopentafluorobenzene (Ipfb), HMTA and 1,4-diodotetrafluorobenzene (Ditfb), were unsuccessful and only yielded bidentate [HMTA]·[Ipfb]_2_ and [HMTA]·[Ditfb]_2_, respectively. The corresponding XB parameters are used for discussions here and their structural data is included in the Supporting Information.

### X-Ray Crystallography

HMTA in complexes **1**–**13** illustrate four types of coordination modes. All the X···N (X = Br, I) distances are below the sum of the Van der Waals radii of respective halogen (Br = 1.58 Å, I = 1.98 Å) and nitrogen (1.55 Å) (Bondi, 1964). Selected bond parameters of X···N in [HMTA]·[dihalogen]_n_ and [HMTA]·[N-haloimide]_n_ and their corresponding X···N normalized interaction ratios (R_XB_) are shown in Tables 1, 2. The X/Y–X···N and (imide)N–X···N interactions are nearly linear with the global ∠X/Y/N–X···N ranging from 173.08(11) to 179.6(9)°.

**Table 1:** Solid-state X-bonding parameters of [HMTA]·[dihalogen]_n_ complexes.

**Complex**	**d(X···N) Å**	**∠X/Y–X···N (**°**)**	**${\text{R}}_{\text{XB}}^{\text{a}}$**
**1**	2.088 (3)	175.24 (9)	0.614
**2**	2.144 (3)	176.65 (9)	0.631
	2.167(3)	175.64 (9)	0.637
**3**	2.402 (5)	173.08 (11)	0.680
**4**	2.272 (5)	174.43 (12)	0.644
**5**	2.474 (4)	174.03 (12)	0.70
	2.486(5)	173.50 (11)	0.704
**6**	2.328 (3)	176.65 (7)	0.659
	2.360(3)	175.75 (7)	0.666

*^a^R_XB_ is defined as [R_XB_ = d_XB_/(X_vdw_+B_vdw_)], where d_XB_ [Å] is the distance between donor (X) and the acceptor atoms (B), X_vdw_ and B_vdw_ are vdW radii [Å] of the corresponding atoms. The vdW radii determined by Bondi (1964) were used to calculate R_XB_ values*.

**Table 2:** Solid-state X-bonding parameters of [HMTA]·[N-haloimide]_n_ complexes.

**Complex**	***d_**1**_* (N–X) Å**	***d_**2**_* (X···N^**′**^) Å**	***d*_1_+$d_{2}^{\text{a}}$ (N···N^**′**^) Å**	**∠N–X···N^′^ (**°**)**	**${\text{R}}_{\text{XB}}^{\text{b}}$**
**7**	1.925 (4)	2.398 (5)	4.317 (7)	173.90 (14)	0.705
	1.919 (4)	2.426 (4)	4.333 (6)	174.54 (14)	0.714
**8**	1.937 (7)	2.388 (7)	4.317 (9)	172.3 (3)	0.702
**9**	1.933 (8)	2.371 (8)	4.304 (11)	177.9 (3)	0.697
	1.924 (7)	2.378 (7)	4.301 (9)	177.5 (3)	0.699
	1.897 (8)	2.411 (9)	4.304 (13)	175.4 (3)	0.709
**10**	1.906 (3)	2.410 (3)	4.308 (5)	179.34 (13)	0.709
**11**	2.143 (8)	2.465 (8)	4.608 (11)	178.3 (3)	0.698
**12**	2.26 (2)	2.29 (2)	4.55 (3)	179.6 (9)	0.649
**13**^c^	2.288 (14)	2.299 (15)	4.58 (2)	175.8 (6)	0.648/0.651

*^a^N is imide nitrogen and N′ is HMTA nitrogen*.

*^b^See Table 1 footnotes for R_XB_ definition*.

c *The structure has been previously reported but the bond parameters are from the current report*.

#### Br–Br···N and Cl–Br···N Halogen Bonds

**Figure 3:**
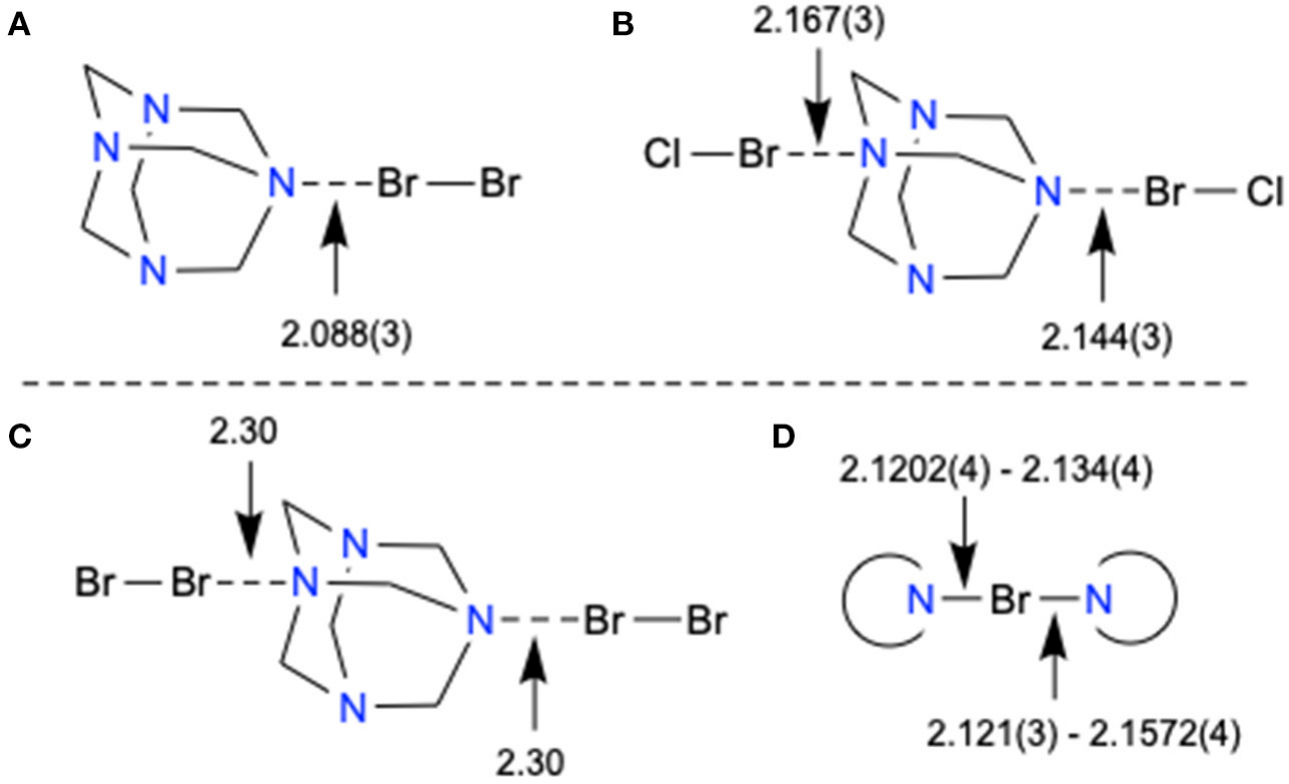
X-Ray structure bond parameters comparison of **(A–C)** [HMTA]·[dihalogen]_n_ and **(D)** non-pyridine [N–Br–N]^+^ XB complexes.

HMTA is monodentate in **1** and bidentate in **2** (Supplementary Figures 1, 2). The Br···N distances varying within a narrow range 2.088(3)−2.167(3) Å are comparable to pyridine-based *3-center-4-electron* [N–Br–N]^+^ XBs [2.086(5)−2.1862(4) Å] (Puttreddy et al., 2019). The Br···N distance of 2.088(3) Å in **1** is shorter than the non-pyridine [N–Br–N]^+^ XBs [2.121(3)−2.1572(2) Å] (Puttreddy et al., 2019), demonstrating an exceptionally tight overlap between nitrogen and bromine (Figure 3A vs. Figure 3D). Complex **2** consists of two crystallographically different Cl–Br···N motifs similar to the reported [HMTA]·[Br_2_]_2_ (Figure 3B vs. Figure 3C) (Eia et al., 1956). The average Br···N distance of **2** is 0.145 Å shorter than [HMTA]·[Br_2_]_2_ due to the electron-withdrawing chloride. The donor σ-hole strength enhancement by a covalently bonded electron-withdrawing atom and the consequent XB distance shortening is in agreement with the literature (Politzer et al., 2017).

#### I–I···N and Cl–I···N Halogen Bonds

Complexes **3** and **4** reveal monodentate coordination manner for HMTA (see Supplementary Figures 3, 4). The Cl–I···N distance of **4** [2.272(5) Å] is remarkably short compared to I–I···N distance of **3** [2.405(5) Å] indicating better e-accepting power of ICl. The monodentate Cl–I···N distance is shorter than ^+^I–N halogen bonds reported for [bis(HMTA)I]^+^${\text{I}}_{3}^{-}$ [2.30(2) Å] (Pritzkow, 1975a) but in the range of non-pyridine based [N–I–N]^+^ XBs [2.198(3)−2.349(18) Å]. In the packing structure, **3** associates via _HMTA_N–I–I···N_HMTA_ halogen bonds at distances of 3.519 Å forming zigzag 1D chains (Supplementary Figure 3). The bidentate structures **5** and **6** are isomorphous (Supplementary Figures 5, 6). The two I···N distances in corresponding structures are comparable to each other. The average I···N distance of **5** is 0.136 Å longer than **6** owing to the stronger e-withdrawing power of chlorine in the latter structure.

**Figure 4:**
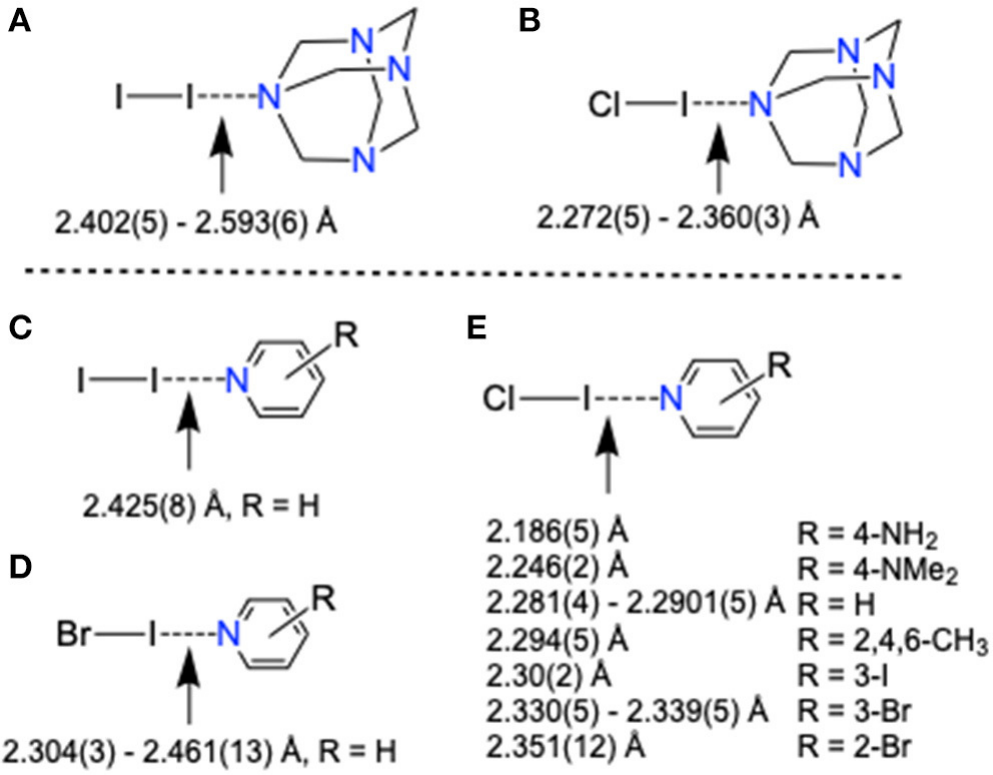
X-Ray structure bond distances comparison of **(A,B)** HMTA-dihalogen and **(C–E)** pyridine-dihalogen complexes.

The average I···N distances of HMTA-I_2_ complexes increase with the increase of HMTA denticity, **3** [2.402(5) Å], **5** [2.480(4) Å], and [HMTA]·[I–I]_3_ [2.593(6) Å] (Tebbe and Nagel, 1995a), suggesting either reduced electrostatic attractive interaction between iodine and nitrogen or simply a consequence of packing forces. Interesting observations were made when I···N distances of HMTA-dihalogen and the pyridine-dihalogens were compared (Figure 4, Table 1, and Supplementary Tables 8–10). The average I···N distances of HMTA-dihalogens follow the order I–I···N_HMTA_ > Cl–I···N_HMTA_ similar to pyridine-dihalogens, I–I···N_py_ > Br–I···N_py_ > Cl–I···N_py_. Unlike HMTA-ICl, pyridine-ICl structures exhibit a broad spectrum of I···N distances (Figure 4B vs. Figure 4E). In Cl–I···N_py_ systems, the substituents' potential to alter the pyridine π-character and N-atom nucleophilicity are responsible factors that can be attributed to the broad range of I···N_py_ distances. This substituent mediated π-electron tunability is not possible in non-aromatic systems such as HMTA.

#### (imide)N–Br···N Halogen Bonds

HMTA is bidentate in **7** and **8**, tridentate in **9**, and tetradentate in **10**. The asymmetric unit of **7** contains one HMTA, two NBS donors, and a chloroform molecule (Supplementary Figure 7). The two Br···N distances in **7** [2.398(5) and 2.426(4) Å] are slightly shorter than our previously reported solvent-free bidentate [HMTA]·[NBS]_2_ [2.414(3) and 2.432(3) Å] (Raatikainen and Rissanen, 2011), suggesting packing forces influence XB parameters in solid-state structures. The average of Br···N distances in **7** is longer by 0.03 Å than the distance in **8** [2.388(7) Å, Supplementary Figure 8] implying the bromine of NBS and NBP have comparable e-accepting power in crystals.

**Figure 5:**
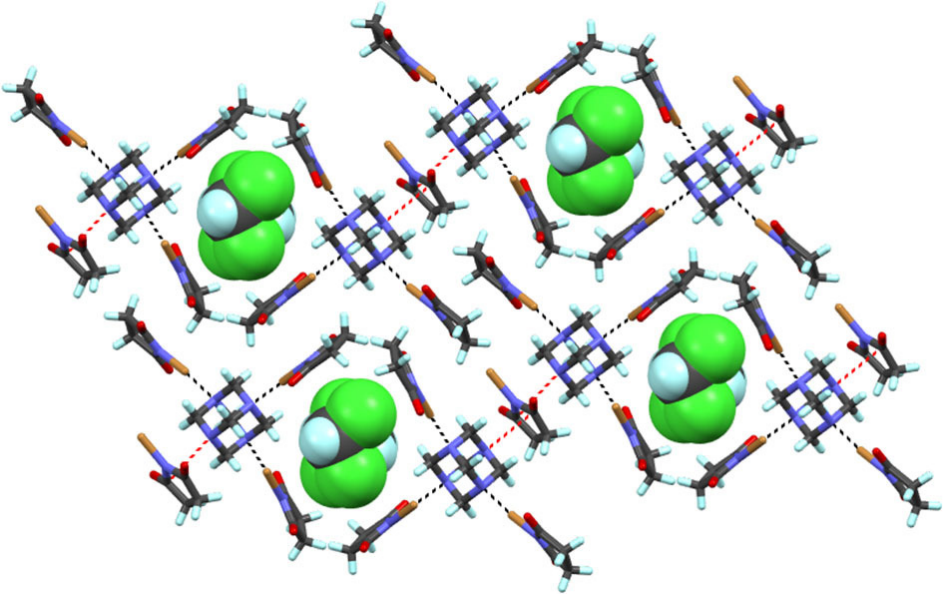
Partial packing view of **9**. Black dotted lines represent Br···N halogen bonds and the red are N···C interactions. CH_2_Cl_2_ guests are in CPK model. The disordered NBS part is omitted for viewing clarity.

**Figure 6:**
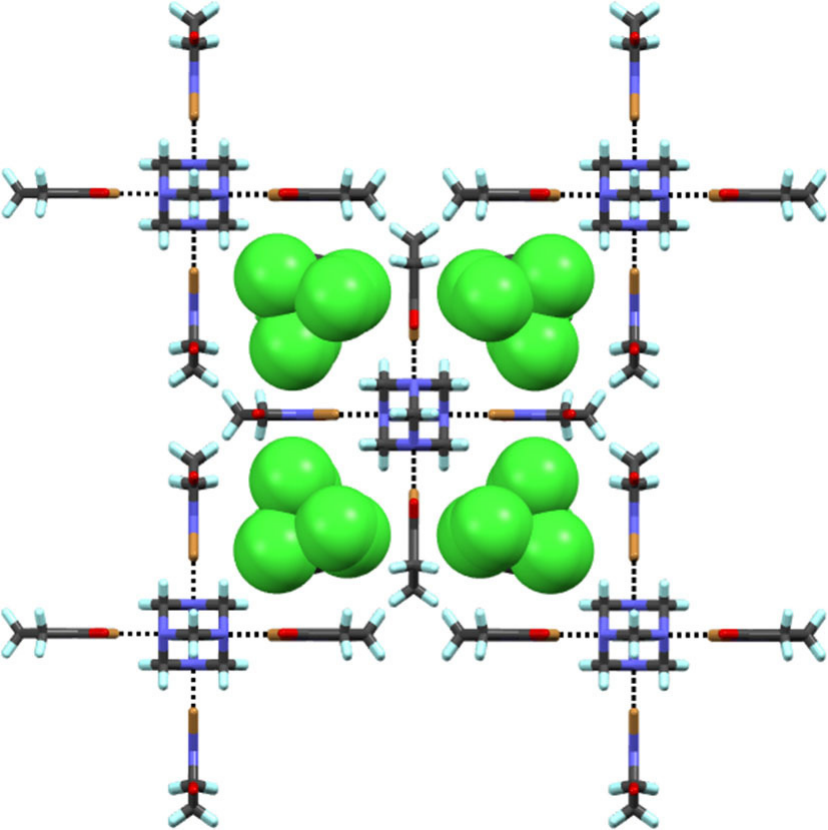
Section of packing view of **10**. Black dotted lines represent Br···N halogen bonds. CCl_4_ guests are in CPK model.

Complex **9** prepared by solvent-assisted manual grinding, contains four crystallographically independent NBS donors of which one NBS does not participate in the X-bonding. Three Br···N distances vary from 2.371(8) to 2.411(9) Å (Table 2). The fourth HMTA nitrogen and the non-halogen-bonded NBS stabilize via N···C interaction [ca. 3.16 Å] as shown in Figure 5 and Supplementary Figure 9. Overall, the 1:4 acceptor:donor units effectively pack through numerous HB interactions to a framework possessing oval shape channels. The channels occupy a total volume of 181 Å^3^/unit cell. The relative channel volume (*rcv*) (Raatikainen and Rissanen, 2012) of **9** is 8.3%, and is the smallest when compared to our earlier XBOF structures (Raatikainen and Rissanen, 2012). Single-crystals of **10** were obtained by hexane diffusion into CCl_4_, which is layered on top of the CHCl_3_ solution containing a 1:4 molar ratio of HMTA:NBS. The white solids at the CCl_4_-CHCl_3_ interface indicate [HMTA]·[NBS]_n_ complexation, and the gradual disappearance suggests potential inclusion of CCl_4_ or CHCl_3_ in crystals. Complex **10** crystallizes in the tetragonal *P*4_2_/*nmc* and the packing structure contains an extended square-grid like channels filled with CCl_4_ molecules (Figure 6), which are structurally similar to CH_2_Cl_2_@[HMTA]·[NIS]_4_ and CCl_4_@[HMTA]·[NIS]_4_ (Raatikainen and Rissanen, 2012). HMTA and NBS form short Br···N halogen bonds [2.401(3) Å] and the distances are comparable to aforementioned structures [HMTA]·[NBS]_n_. Their channels occupy a volume of 934.3 Å^3^/unit cell, and *rcv* of 41.5% is the largest value of all [HMTA]·[NIS]_4_ XBOFs (Raatikainen and Rissanen, 2012).

#### (imide)N–I···N Halogen Bonds

**Figure 7:**
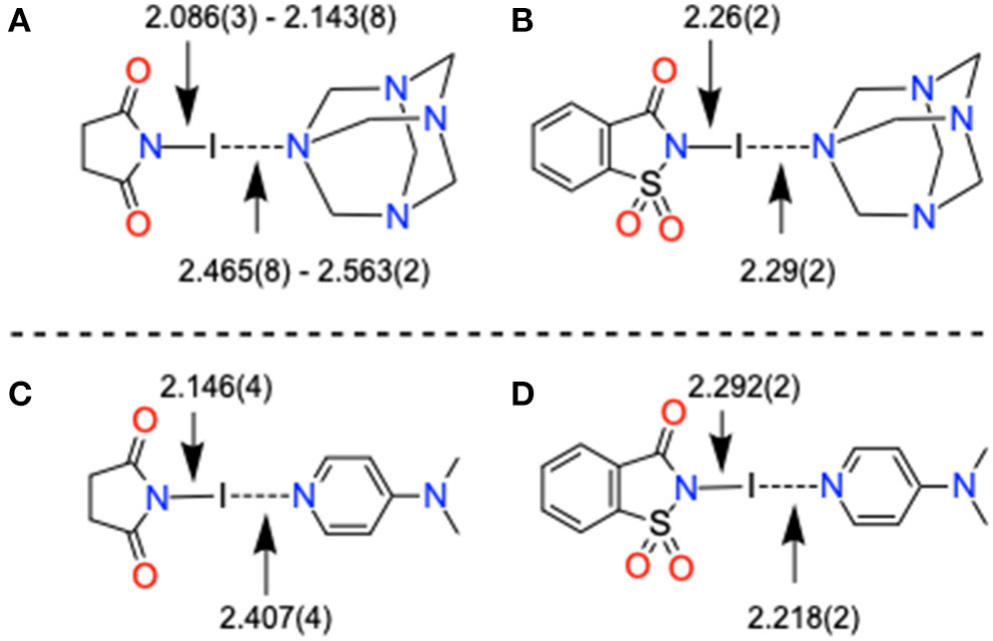
X-Ray structure bond parameters comparison of **(A**,**B)** [HMTA]·[N-haloimide]_n_ and **(C**,**D)** pyridine-N-haloimide complexes.

Complex **11** contains bidentate HMTA, and the I···N distances [2.465(8) Å, Supplementary Figure 11] are shorter compared to C–I···N of bidentate structures, [HMTA]·[Ipfb]_2_ [2.799 (2) and 2.771(2) Å, Supplementary Figure 12], [HMTA]·[diiodobenzene]_2_ [2.981(2) Å] (Szell et al., 2019), [HMTA]·[Ditfb]_2_ [2.816(2) Å, Supplementary Figure 13], and [HMTA]·[1,3,5-triiodoperfluorobenzene]_2_ [2.879(5) and 2.864(4) Å] (Syssa-Magalé et al., 2014). The short I···N distance between HMTA and NIS is due to the high e-accepting power of NIS iodine. 4-(Dimethylamino)pyridine (DMAP) is one of the strongly σ-donating ligands in the aromatic N-heterocycles family and is known to form strong halogen bonds with N-iodoimides (Makhotkina et al., 2015). The average (imide)N–I [2.105 Å Å] distance of [HMTA]·[NIS]_n_ is close to the corresponding distance reported for [DMAP]·[NIS], as shown in Figures 7A,C (for bond parameters, see Table 2 and Supplementary Table 6). The average I···N(HMTA) of [HMTA]·[NIS]_n_ is 0.121 Å longer than I···N(DMAP) distances of [DMAP]·[NIS]. However, both the (imide)N–I [2.26(2) Å] and I···N(HMTA) distances [2.29(2) Å] of **12** are remarkably close to [DMAP]·[NISac] [2.292(2) and 2.218(2) Å] (Figures 7B,D). Complex **13** bond parameters are similar to the reported structure (Pritzkow, 1975a). The two ^+^I–N bond distances of **13** are 2.288(14) and 2.299(15) Å, and appear within 0.02 Å of the corresponding distances in the reported structure (Supplementary Figure 15).

## Computational Studies

### Molecular Electrostatic Potentials (MEP)

**Figure 8:**
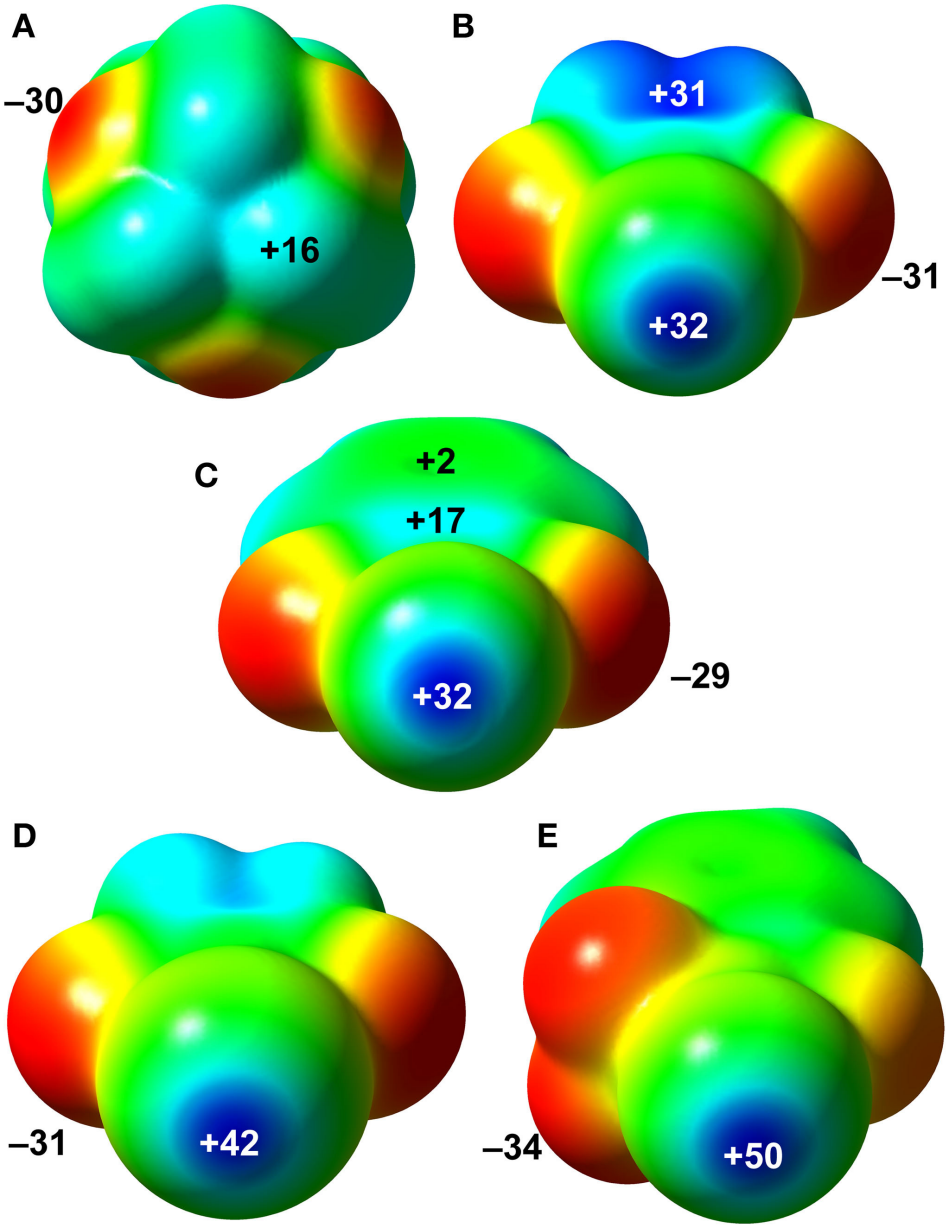
MEP surfaces of HTMA **(A)**, NBS **(B)**, NBP **(C)**, NIS **(D)**, and NISac **(E)**. The MEP values are shown at selected points are given in kcal/mol. Positive potential values are blue, and the negative values in red.

In order to evaluate the donor-acceptor abilities of HMTA and N-haloimides, MEP were mapped onto their respective Van der Waals surfaces as depicted in Figure 8. The most negative potential, located at the HMTA N-atoms (V_S, min_, −30 kcal/mol), is comparable to values estimated at the O-atoms of NBS (−31 kcal/mol) and NBP (−29 kcal/mol). A V_S, max_ of magnitude +16 kcal/mol is associated with the –CH_2_- protons adjacent to the *sp*^3^ N-atom. In NBS, the V_S, max_ at the bromine σ-hole and the five-member ring-centroid are similar (Figure 8B). The V_S, max_ of NBP bromine σ-hole is the same as the NBS; however, the V_S, max_ values over its five- and six-membered ring centroids are significantly smaller (Figure 8C, +17 and +2 kcal/mol). Compared to the aforementioned donor σ-hole strengths, the NIS and NISac iodine σ-holes V_S, max_ values +42 and +50 kcal/mol are significantly larger. The NIS five-membered ring-centroid V_S, max_ value (+30 kcal/mol) is slightly smaller than in NBS but larger than in NBP. To our surprise, the electron-withdrawing –SO_2_ group of NISac could only render a positive potential of +7 kcal/mol at the six-membered ring-centroid. The global MEP analysis suggests that the nucleophilic and electrophilic sites of HMTA and NBS/NIS molecules have equal propensity to form Br···N, C–H···N, and C–H···O=C interactions, which is in good agreement with packing forces discussed in the XBOF structures (Raatikainen and Rissanen, 2012).

### Additivity Interaction Energy

**Figure 9:**
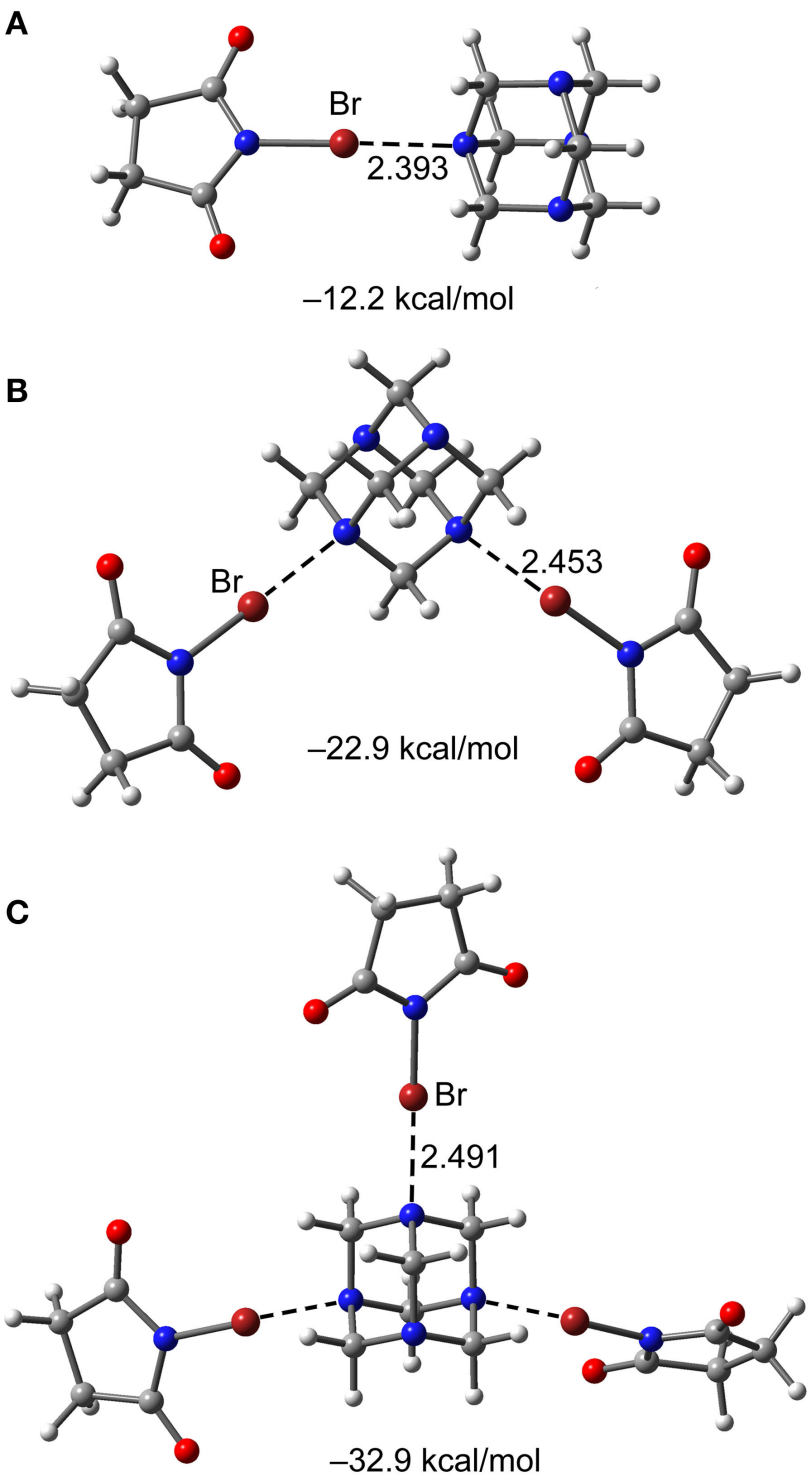
Interaction energies of the 1:1 **(A)**, 1:2 **(B)**, and 1:3 **(C)** [HTMA]·[NBS] complexes.

The additivity interaction energy, defined as the interaction energy enhancement or reduction of a (imide)X···N motif in a 1:1 complex when donors are successively added to HMTA, are estimated for complexes [HTMA]·[NBS]_1−3_. The RI-MP2/def2-TZVP optimized bond distances and interaction energies of the 1:1, 1:2, and 1:3 complexes of [HTMA]·[NBS]_n_ are shown in Figure 9. The XB interaction energy in the 1:1 complex is −12.2 kcal/mol and has progressively reduced to −11.45 kcal/mol in 1:2 complex to −10.97 kcal/mol in 1:3 complex. The energy analysis is consistent with the N···Br equilibrium distance that gradually increases from 1:1 to 1:3 complex.

### Interaction Energies

**Figure 10:**
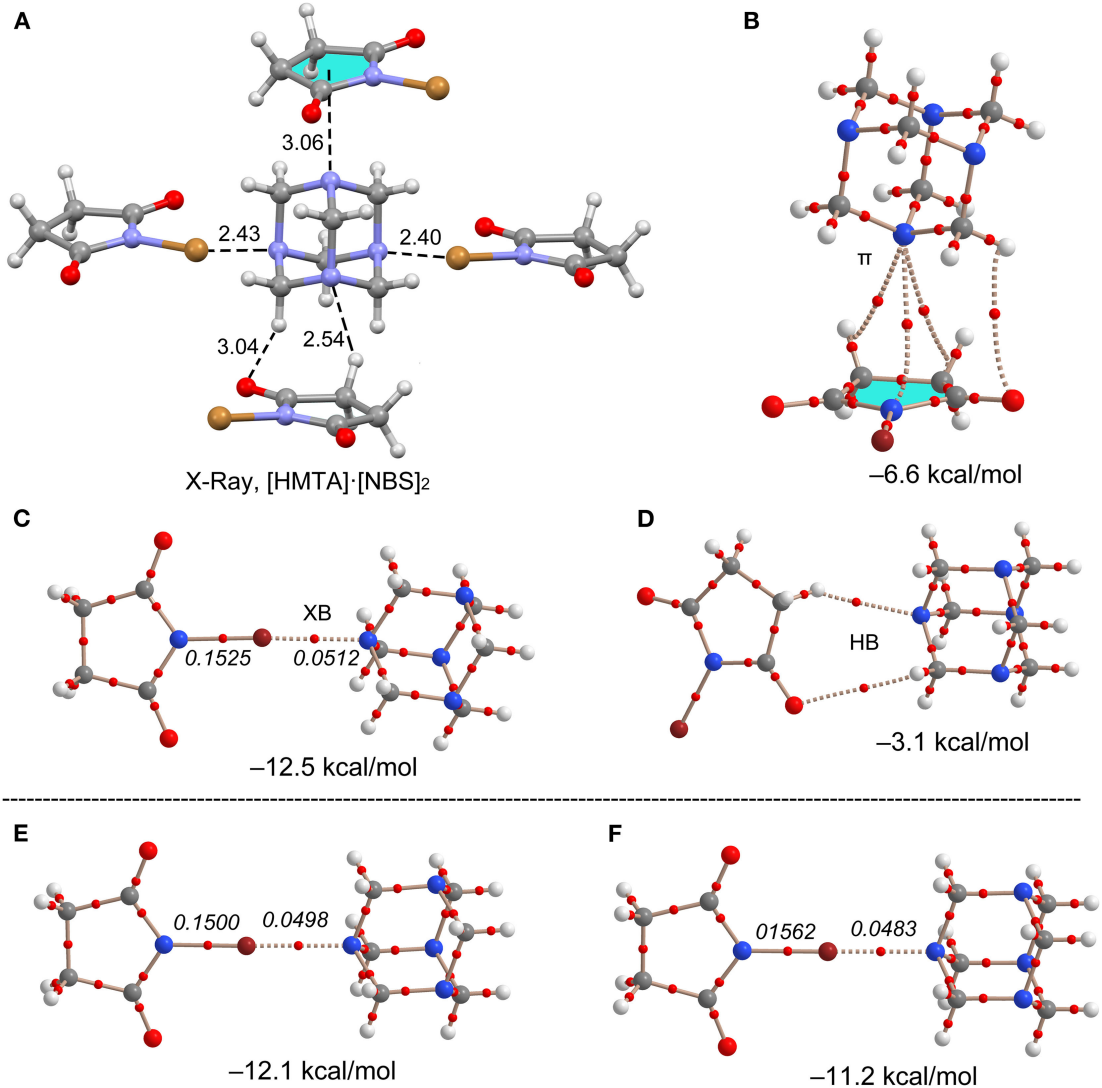
**(A)** Asymmetric unit view of **7**. **(B**–**D)** Different theoretical models, AIM analyses (BCPs in red), and interaction energies for two representative dimers of **7**. XB Dimers of **(E) 9** and **(F) 10**. Distances in Å. The values in italics correspond to the ρ(r) values at the bond CP in a.u.

All interaction energies (ΔE_int_) were estimated by using their corresponding X-ray crystal structure coordinates. The calculated ΔE_int_ values range from −11.2 to −12.5 kcal/mol for Br···N motifs. The ΔE_int_ values decrease when the HMTA denticity increases, −12.5 kcal/mol for **7**, −12.1 kcal/mol for **9**, and −11.2 kcal/mol for **10**, and the results are in good agreement with the additivity analysis. The ΔE_int_ value of **7** is stronger than NBS···N_Py_ (−9.2 kcal/mol) (Stilinović et al., 2017), and Br···N interactions between NBS and substituted pyridines (−6.7 to 11.3 kcal/mol) (Stilinović et al., 2017). These results indicate that the *sp*^3^ N-atom lone-pair overlaps better with NBS bromine σ-hole compared to the aromatic *sp*^2^ nitrogen. In all the Br···N motifs, the charge density values ρ(r) for N–Br covalent bond and Br···N non-covalent bond are significantly different, indicating the absence of a shared-shell character (see Figure 10). Similarly, a contrary covalent character has been recently described for NBSac···N_Py_ structures (Aubert et al., 2017). Similar to the interaction energies, a decreasing trend for charge density ρ(r) at the bond critical point (BCP) was observed for **7**, **9**, and **10** complexes.

In contrast to the same σ-hole strengths of NBS and NBP, the Br···N halogen bonds interaction strengths of **8** (−14.0 kcal/mol) are slightly larger than in **7** (−12.5 kcal/mol). This agrees with the well-known fact that the fused six-membered ring in NBP removes electron density from the five-membered ring, consequently making the bromine more electrophilic. The ΔE_int_ values of ancillary interactions, for example, N···π(ring-centroid) and C–H···N hydrogen bond contacts in **7**, are estimated to understand their interaction strengths relative to Br···N motifs (see Figure 10). The ΔE_int_ of N···π (−6.6 kcal/mol) and C–H···N (−3.1 kcal/mol) contacts are weaker than XBs (−12.5 kcal/mol). The presence of the BCPs and bond paths of their connecting atoms are other evidences for N···π and C–H···N contacts. This suggests that weak and moderately strong HBs, that originate from donor-acceptor components' electron-rich and deficient sites, are inevitable and may contribute to the XB stabilization energy.

**Figure 11:**
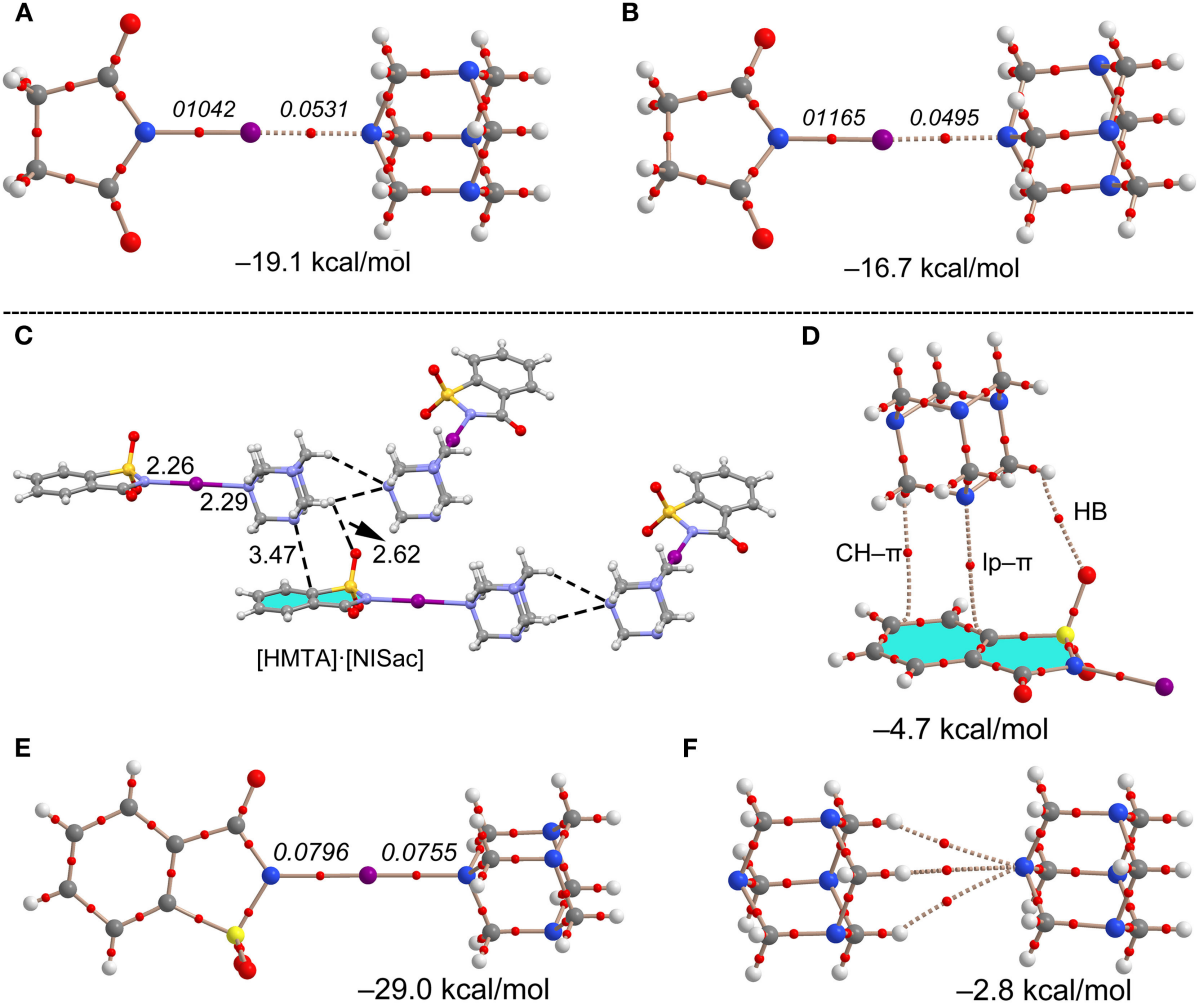
Theoretical and AIM analysis of dimer models of **7 (A)** and **9 (B)**. Partial molecular packing view of **12 (C)**, and corresponding theoretical and AIM analysis of dimeric structures, lp–π and C–H···π **(D)**, halogen **(E)**, and trifurcated H-bond **(F)**. Distances in Å. The values in italics correspond to the ρ(r) values at the bond CPs in a.u.

The I···N interaction energies of **11** (−19.1 kcal/mol) and [HMTA]·[NIS]_4_ (−16.7 kcal/mol) are larger than corresponding bromine structures, and twice the energy of C–I···N contacts (−8.4 kcal/mol) in [HMTA]·[Iodopentafluorobenzene]_2_ (see Supplementary Figure 16). The ΔE_int_ and the ρ(r) values at the BCP of **11** are somewhat larger than [HMTA]·[NIS]_4_, which agrees with the additivity analysis. Complex **12** involving NISac has the largest interaction energy of all the (imide)I···N_HMTA_ halogen bonds (−29.0 kcal/mol) owing to larger iodine σ-hole strength in NISac (+50 kcal/mol). The ρ(r) values at the BCPs of I···N halogen bond, similar to N–I covalent indicating a degree of covalency with shared-shell character, is remarkable. This agrees with the N–I and I···N bonds covalent character discussion in [DMAP]·[NISac] (Makhotkina et al., 2015). In order to evaluate ancillary interactions in the packing structure, ΔE_int_ values are estimated of representative C–H···O=S and C–H···π dimers shown in Figure 11. The ΔE_int_ values of the lp–π and C–H···π interactions are −4.7 and −2.8 kcal/mol. The C–H···π interactions exhibit a modest interaction energy (−2.8 kcal/mol) and are comparable to HB energies observed in **7**. The AIM analysis reveals that C–H···O=S and C–H···π interactions may contribute to the formation of I···N_HMTA_ interactions.

## Conclusion

In summary, we investigated Y–X···N (X = Br, I and Y = N, Cl, Br, I) halogen bonds in 13 X-ray crystal structures obtained from HMTA and N-haloimides. Two complexes of HMTA with iodoperfluorobenzene and 1,4-diiodotetrafluorobenzene consisting of C–I···N halogen bonds were also prepared, and their solid-state structures were studied for comparison purposes. The Y–X···N distances depend on the nature of Y-atom and the donor scaffold. The Br/Cl–Br···N [2.088(3)−2.167(3) Å] are shorter than (imide)N–Br···N [2.371(8)−2.426(4) Å] halogen bonds. In contrast, the I/Cl–I···N [2.328(3)−2.486(5) Å] tend to be longer when compared to (imide)N–I···N [2.29(3) and 2.502(10) Å] halogen bonds. The shortest I···N [2.29(3) Å] distance between HMTA and *N*-iodosaccharin even approaches the reported *3*-*center*-*4*-*electron* halogen bonds of [(HMTA)N–I–N(HMTA)]^+^ [2.288(14) and 2.299(15) Å]. The scope of halogen-bonded organic frameworks (XBOFs), previously accessed by (imide)I···N using a 1:4 [HMTA]:[NIS] building block, is expanded to (imide)Br···N halogen-bonded 1:3 [HMTA]:[NBS] and 1:4 [HMTA]:[NBS] structures. Different from (imide)I···N XBOFs, channel shape adaptability is achieved through HMTA tridentate and tetradentate coordination modes. DFT based MEPs provided us with important experimental insights into the nature of donor-donor and donor-acceptor interactions. Donors, such as NBS/NIS possessing σ-hole (V_S, max_) and C–H acidic proton (V_S, min_) values, have high probabilities to form XBOFs via (imide) X···N halogen bonds and orthogonal C–H···O=C hydrogen bonds. The lack of acidic *sp*^3^ C–H protons, like in NBP, encourage π-π and other hydrogen bond interactions obstructing the formation of the desired 1:4 ratio [HMTA]:[NBP] and eventually affect XBOFs' self-assembly processes. In terms of DFT interactions energies, the (imide)N–X···N(HMTA) halogen bonds varying from −11.2 to −12.5 kcal/mol for X = Br, and −8.4 to −29.0 kcal/mol for X = I, are stronger than corresponding (imide)N–X···N(pyridines) halogen bonds. A comprehensive solution NMR study on [HMTA]·[N-haloimide]_n_ complexes, optimization of crystallization conditions to synthesize XBOFs using other HMTA-imide combinations, and post-synthetic solvent exchange process of (imide)N–Br···N XBOFs are currently under investigation in our laboratory.

## Data Availability Statement

The datasets presented in this study can be found in online repositories. The names of the repository/repositories and accession number(s) can be found in the article/Supplementary Material.

## Author Contributions

RP was responsible for supervision, methodology development, manuscript preparation, and SCXRD analysis. All halogen bond complexes, except **12**, for X-ray crystallography, done in JYU, were prepared by GA, **12** was prepared by LG. Both JR and GP were external thesis supervisors for GA and LG, respectively. AF and AB were responsible for computational studies. KR was responsible for proofreading the final manuscript version. All authors have read and agreed to the published version of the manuscript.

## Conflict of Interest

The authors declare that the research was conducted in the absence of any commercial or financial relationships that could be construed as a potential conflict of interest.
