# Supplementary material for: Short X···N Halogen Bonds With Hexamethylenetetraamine as the Acceptor
Source: Front Chem. 2021 Apr 29;9:623595. doi: 10.3389/fchem.2021.623595 (PMC8116742; doi:10.3389/fchem.2021.623595)
Supplement: Supplementary file 1 [file Data_Sheet_1.docx]

Supplementary Material

# General Information

All solvents employed for synthesis and crystallization experiments are commercially purchased and were used as received without any purification. The following solvents and chemicals are used as purchased without further purification: hexamethylenetetramine (99.5 %, Sigma Aldrich), iodine (98 %, TCI), N-chlorosuccinimide (98 %, Sigma Aldrich), N-bromosuccinimide (99 %, Sigma Aldrich), N-bromophthalimide (95 %, Sigma Aldrich), N-iodosuccinimide (98%, TCI), N-iodosaccharin (98%, TCI), diiodotetrafluorobenzene (98 %, Sigma Aldrich) iodopentafluorobenzene (95 %, TCI), acetone (99.9 %, VWR), acetonitrile (99.9 %, Fisher), chloroform (99.9 %, Fisher), dichloromethane (99.9 %, VWR), nitromethane (95%, J.T. Baker), 1,2-nitropropane (96 %, J.T. Baker), tetrachloromethane (≥ 99.5 %, VWR), and toluene (≥ 99.5 %, VWR).

**Abbreviations:** Hexamethylenetetramine = HMTA; Iodine = I_2_; N-Bromosuccinimide = NBS; N-Chlorosuccinimide = NCS; N-Iodosuccinimide = NIS; N-Bromophthalimide = NBP; N-Iodosaccharin = NISac; Iodopentafluorobenzene = Ipfb; Diiodotetrafluorobenzene = Ditfp; Acetonitrile = ACN; chloroform = CHCl_3_; Dichloromethane = CH_2_Cl_2_.

# Synthesis of halogen bond complexes

**2.1 Synthesis of [HMTA]**･**[Br_2_]** (**1**)

**Method 1**: To HMTA (20.0 mg, 0.143 mmol) in ACN (10 mL), was added a colourless solution of NBS (101.80 mg, 0.572 mmol) dissolved in ACN (10 mL). The white precipitates were filtered, hexane diffusion into the yellow colour filtrate solution gave orange colour crystals.

**Method 2**: Solid NBS (101.80 mg, 0.572 mmol) was added to HMTA (20.0 mg, 0.143 mmol) dissolved in chloroform (20 mL). In three different experiences, solvent 2 and/or solvent 3 were layered over the CHCl_3_ solution at the room temperature (See Table 1). The corresponding final solutions were subjected to hexane vapours to give orange colour crystals suitable for X-ray crystallography. [HMTA]･[Br_2_] (**1**) is the major product in all three experiments.

**Table S1**.List of solvents for the synthesis of **1**.

| Entry | HMTA+NBS in  Solvent 1 | Solvent 2 | Solvent 3 | Volume ratio of solvents 1:2:3 |
| --- | --- | --- | --- | --- |
| 1 | CHCl_3_ | 2-nitropropane | -- | 1:1 |
| 2 | CHCl_3_ | CCl_4_ | 2-nitropropane | 1:1:1 |
| 3 | CHCl_3_ | CCl_4_ | Nitromethane | 1:1:1 |

**2.2 Synthesis of [HMTA]**･**[BrCl]_2_** (**2**)

**Method 1:** To HMTA (9.0 mg, 0.064 mmol) in ACN (10 mL), added solid NBS (11.4 mg, 0.064 mmol). The solution is hand shaken to dissolve all the components. To this yellow colour solution, added a light yellow colour solution of NCS (8.6 mg, 0.064 mmol) dissolved in ACN (4 mL). The final solution is left at room temperature to evaporate slowly to give orange colour crystals. Note: Same experiment repeated using 2:2:1 and 4:4:1 molar ratios of HMTA:NIS:NCS resulted in 1:2 complex, [HMTA]･[BrCl]_2_.

**Method 2:** To NBS (50.54 mg, 0.284 mmol) dissolved in ACN (1 mL), NCS (37.92 mg, 0.284 mmol) dissolved in 1 mL of ACN was added at room temperature. The resultant NBS:NCS solution was added dropwise to HMTA (10 mg, 0.071 mmol) dissolved in ACN (2.5 mL) at room temperature. The final colourless solution was left at room temperature to slowly evaporate to give orange colour crystals.

**2.3 Synthesis of [HMTA]**･**[I_2_]** (**3**)

**Method 1:** To HMTA (107.0 mg, 0.763 mmol) in ACN (20 mL), was added a light purple colour solution of NIS (188.9 mg, 0.840 mmol) dissolved in ACN (40 mL). The white precipitates were filtered, hexane diffusion into the purple colour filtrate gave orange colour crystals.

**Method 2:** To HMTA (51.0 mg, 0.364 mmol) in ACN (20 mL), was added a light purple colour solution of NIS (90.0 mg, 0.40 mmol) dissolved in ACN (40 mL). Acetonitrile was added dropwise to dissolve white precipitates and hexane diffusion into the purple colour filtrate gave orange colour crystals.

**2.4 Synthesis of [HMTA]**･**[ICl]** (**4**)

**Method 1:** To HMTA (10.5 mg, 0.075 mmol) in ACN (10 mL), added solid NIS (16.9 mg, 0.075 mmol). The solution is hand shaken to dissolve all the components. To this purple colour solution, added a light yellow colour solution of NCS (10.0 mg, 0.075 mmol) dissolved in ACN (4 mL). The final solution is left at room temperature to evaporate slowly to give orange-purple colour crystals.

**Method 2:** The 1:1 mixture of NIS (45.0 mg, 0.20 mmol) of NCS (26.7 mg 0.20 mmol) dissolved in ACN (4 mL) is added dropwise to HMTA (28.0 mg, 0.20 mmol) dissolved in ACN (3 mL). The formed precipitates were filtered, and the purple-orange filtrate is left at room temperature to evaporate slowly to give orange colour crystals.

**2.5 Synthesis of [HMTA]**･**[I_2_]_2_** (**5**)

To HMTA (10 mg, 0.071 mmol) dissolved in chloroform (4 mL), NIS (64 mg, 0.285 mmol) dissolved in chloroform (7 mL) was added at room temperature. The final purple-red coloured solution was left at room temperature protected from light, and subjected to slow evaporation to yield orange-purple colour crystals.

**2.6 Synthesis of [HMTA]**･**[ICl]_2_** (**6**)

Two solutions were prepared, 63.89 mg of NIS (0.284 mmol) dissolved in ACN (2 mL) and 37.92 mg of NCS (0.284 mmol) dissolved in ACN (2 mL). The NIS-NCS mixture was added dropwise to HMTA (10.0 mg, 0.071 mmol) dissolved in ACN (2.5 mL). Precipitation formation observed. The solution was filtered and left to evaporate slowly at room temperature to give orange colour crystals.

**2.7 Synthesis of [HMTA]**･**[NBS]_2_** (**7**)

**Method 1**: To HMTA (20 mg, 0.143 mmol) dissolved in chloroform (22 mL), solid NBS (101.80 mg, 0.572 mmol) were added in portions. Solid NBS dissolution require exhaustive shaking. The clear yellow solution was subjected to hexane vapours diffusion to yield single-crystals for X-ray crystallography. Various solvents were tested for their influence on the growth of single crystals as shown in Table S2. One or two more solvents are layered separately over the clear yellow solution followed by hexane vapours diffusion. In all crystallization experiments of Table 2, formation of **7** is the major product.

**Table S2**. List of solvents for the synthesis of **7**.

| Entry | HMTA+NBS in  Solvent 1 | Solvent 2 | Solvent 3 | Volume ratio of solvents 1:2:3 |
| --- | --- | --- | --- | --- |
| 1 | CHCl_3_ | CH_2_Cl_2_ | -- | 1:1 |
| 2 | CHCl_3_ | CCl_4_ | CH_2_Cl_2_ | 1:1:1 |
| 3 | CHCl_3_ | CCl_4_ | CH_2_Cl_2_ | 1:0.1:0.9 |
| 4 | CHCl_3_ | CCl_4_ | CH_2_Cl_2_ | 1:0.2:0.8 |
| 5 | CHCl_3_ | CCl_4_ | Toluene | 1:1:1 |
| 6 | CHCl_3_ | CCl_4_ | Toluene | 1:0.1:0.9 |
| 7 | CHCl_3_ | CCl_4_ | Toluene | 1:0.2:0.8 |
| 8 | CHCl_3_ | CCl_4_ | ACN | 1:1:1 |
| 9 | CHCl_3_ | CCl_4_ | Acetone | 1:1:1 |

**Method 2**: 5.0 mg of HMTA (0.036 mmol), 25.63 mg of NBS (0.144 mmol), and three drops of dichloromethane placed in a mortar were finely ground. The yellow powder was transferred into a 20 mL vial and added chloroform (7 mL). The mixture was exhaustively shaken to dissolve the components and the resultant yellow solution was subjected to hexane vapor diffusion.

**2.8 Synthesis of [HMTA]**･**[NBP]_2_** (**8**)

White powder precipitates and small crystals started to form immediately after NBP (32.54 mg, 0.144 mmol) dissolved in ACN (5 mL) dropwise addition to HMTA (5.0 mg, 0.036 mmol) dissolved in warm ACN (5 mL). The solution was allowed to slowly evaporate at room temperature to give colourless crystals.

**2.9 Synthesis of [HMTA]**･**[NBS]_4_** (**9**)

5.0 mg of HMTA (0.036 mmol), 25.63 mg of NBS (0.144 mmol) and three drops of either tetrachloromethane or toluene or ACN were placed in a mortar and were finely ground. The final yellow powder was dissolved in 5 mL of CH_2_Cl_2_ by exhaustive shaking. The colourless solution left at room temperature was subjected to hexane vapour diffusion to give colourless crystals.

**2.10 Synthesis of [HMTA]**･**[NBS]_4a_** (**10**)

To HMTA (20 mg, 0.143 mmol) dissolved in chloroform (20 mL), solid NBS (101.80 mg, 0.572 mmol) were added in portions and, solids were dissolved by using exhaustive manual shaking. Then, 20 mL of CCl_4_ was gently layered using a Pasteur pipette. White solids that formed at the interface between the two solvents gradually disappeared giving a homogeneous solution. The solution subjected to hexane vapor diffusion gave colourless crystalline needles suitable for X-ray analysis.

**2.11 Synthesis of [HMTA)]**･**[NIS]_2_** (**11**)

**Method 1**: ACN (13 mL) was carefully layered over HMTA (5.0 mg, 0.036 mmol) dissolved in chloroform (13 mL). A third solution, NIS (32.4 mg, 0.144 mmol) dissolved in dichloromethane (13 mL) was layered over ACN. The resulting solution characterized by two distinguishable layers was subjected to hexane vapour diffusion to give colourless crystals.

**Method 2**: The above procedure was followed by using solvents shown in Table 3, and all experiments yielded single-crystals of **11** as the major product for crystal structure analysis.

**Table S3**. List of solvents for the synthesis of **11**

| Entry | HMTA in  Solvent 1 | Solvent 2 | NIS in  Solvent 3 | Volume ratio of solvents 1:2:3 |
| --- | --- | --- | --- | --- |
| 1 | CHCl_3_ | ACN | Acetone | 1:1:1 |
| 2 | ACN | CH_2_Cl_2_ | ACN | 1:1:1 |
| 3 | ACN | Toluene | ACN | 1:1:1 |
| 4 | ACN | CHCl_3_ | ACN | 1:1:1 |
| 5 | ACN | CCl_4_ | ACN | 1:1:1 |

**2.13 Synthesis of [HMTA]**･**[NISac]_n_**

**1)** To HMTA (10 mg, 0.071 mmol) dissolved in ACN (4 mL), added solid NISac (46.3 mg, 0.150 mmol) at room temperature. The pale purple solution left at room temperature to give colourless crystals of complex [HMTA]･[NISac] (**12**).

2) To HMTA (11.2 mg, 0.080 mmol) dissolved in 1:1 ACN:CHCl_3_ (4 mL), added NISac (202.5 mg, 0.655 mmol) dissolved in acetone (5 mL) at room temperature. The purple colorless solution was subjected to slow evaporation to give brown crystals of [bis(HMTA)I]I_3_ (**13**).

**2.12 Synthesis of [HMTA]**･**[Ipfb]_2_**

To HMTA (5.0 mg, 0.036 mmol) dissolved in ACN (1 mL), added Ipfb (84.66 mg, 0.288 mmol) at room temperature. The colorless solution was subjected to slow evaporation to give colourless crystals.

**2.13 Synthesis of [HMTA]**･**[Ditfb]_2_**

In all three methods, the final solutions were colourless and the resulted complex is [HMTA]･[Ditfb]_2_. Slow evaporation method is followed to obtain colourless crystals for X-ray crystal structure analysis.

**Method 1**: Solid HMTA (23.0 mg, 0.164 mmol) and Ditfb (33.0 mg, 0.082 mmol) were added to 5 mL of ACN at room temperature and hand shaken to dissolve all the substrates.

**Method 2**: To HMTA (10.5 mg, 0.075 mmol) dissolved in ACN (2 mL), was added solid Ditfb (60.2 mg, 0.150 mmol) at room temperature.

**Method 3**: To HMTA (7.0 mg, 0.050 mmol) dissolved in ACN (2 mL), was added Ditfb (82.3 mg, 0.205 mmol) at room temperature.

## 3. X-Ray crystallography

All data were measured using (a) a single-source Rigaku SuperNova diffractometer equipped with an Atlas detector and an Atlas EoS CCD detector using mirror-monochromated Mo-Kα radiation (λ = 0.71073 Å). Data collection and reduction for all complexes were performed using the program CrysAlisPro (Rigaku, 2017) and Gaussian face-index absorption correction method was applied (Rigaku, 2017); (b) a Bruker-Nonius KappaCCD diffractometer with an APEX-II detector with graphite-monochromatized Mo-Kα (λ = 0.71073 Å) radiation. Data collection and reduction were performed using the program COLLECT (Hooft R. W. W., 1998) and HKL DENZO AND SCALEPACK, (Otwinowski and Minor, 1997) respectively, and the intensities were corrected for absorption using SADABS (Sheldrick, 1996). All structures were solved with Direct Methods (SHELXS) (Sheldrick, 2008, 2015) and refined by full-matrix least squares based on F^2^ using the OLEX2 software (Dolomanov et al., 2009), which utilizes the SHELXL-2013 (Sheldrick, 2008, 2015). Non-hydrogen atoms were assigned anisotropic displacement parameters unless stated otherwise. Hydrogen atoms were placed in idealized positions and included as riding. Isotropic displacement parameters for all H atoms were constrained to multiples of the equivalent displacement parameters of their parent atoms with Uiso(H) = 1.2 Ueq (parent atom). In addition, restraints (e.g. DFIX) were applied for disordered systems. Positional disorders were refined to the respective two split positions (For more details, see cifs), with the sum of the site occupancies of both alternative positions constrained to unity. The X-ray single crystal data and experimental details and CCDC numbers (2041018-2041032) are given below.

1) Crystal data for [HMTA]·[Br_2_] (**1**): CCDC - 2041018, C_6_H_12_Br_2_N_4_, M = 300.02 gmol^-1^, orange colour block, 0.4 × 0.27 × 0.09 mm^3^, monoclinic, space group *P*2_1_/*c*, a = 5.9077(2), b = 13.7052(4) Å, c = 11.8940(4) Å, α = 90°, β = 99.273(3)°, γ = 90°, V = 950.43(5) Å^3^, Z = 4, D_calc_ = 2.097 gcm^3^, F(000) = 584, µ = 8.482 mm^-1^, T = 120 K, θ_max_ = 25.248°, 4698 total reflections, 1516 with Io > 2σ(Io), R_int_ = 0.0456, 1717 data, 109 parameters, 0 restraints, GooF = 1.083, R_1_ = 0.0328 and wR_2_ = 0.0761 [Io > 2σ(Io)], R_1_ = 0.0385 and wR_2_ = 0.0787 (all reflections), 0.508 < d∆ρ < –0.707 eÅ^3^.

2) Crystal data for [HMTA]·[BrCl]_2_ (**2**): CCDC - 2041019, C_6_H_12_Br_2_C_l2_N_4_, M = 370.92 gmol^-1^, orange colour block, 0.22 × 0.16 × 0.13 mm^3^, monoclinic, space group *P*2_1_*/c*, a = 6.0091(12), b = 14.422(3) Å, c = 13.655(3) Å, α = 90°, β = 92.17(3)°, γ = 90°, V = 1182.5(4) Å^3^, Z = 4, D_calc_ = 2.083 gcm^3^, F(000) = 720, µ = 7.277 mm^-1^, T = 170 K, θ_max_ = 25.248 °, 7988 total reflections, 1790 with Io > 2σ(Io), R_int_ = 0.0406, 2144 data, 127 parameters, 0 restraints, GooF = 1.036, R_1_ = 0.0296 and wR_2_ = 0.0649 [Io > 2σ(Io)], R_1_ = 0.0413 and wR_2_ = 0.0694 (all reflections), 0.595 < d∆ρ < –0.359 eÅ^3^.

3) Crystal data for [HMTA]·[I_2_] (**3**): CCDC- 2041020, C_6_H_12_I_2_N_4_, M = 394.00 gmol^-1^, orange colour plate, 0.19 × 0.17 × 0.10 mm^3^, orthorhombic space group *Cmc2_1_*, a = 9.2388(18), b = 7.6121(15) Å, c = 15.423(3) Å, α = 90°, β = 90°, γ = 90°, V = 1084.6(4) Å^3^, Z = 4, D_calc_ = 2.413 gcm^3^, F(000) = 1152, µ = 5.759 mm^-1^, T = 170 K, θ_max_ = 28.870 °, 14699 total reflections, 1483 with Io > 2σ(Io), R_int_ = 0.0344, 1505 data, 64 parameters, 1 restraints, Flack parameter = 0.350(14), GooF = 1.097, R_1_ = 0.0160 and wR_2_ = 0.0368 [Io > 2σ(Io)], R_1_ = 0.0164 and wR_2_ = 0.0369 (all reflections), 0.449 < d∆ρ < –0.537 eÅ^3^.

4) Crystal data for [HMTA]·[ICl] (**4**): CCDC - 2041021, C_6_H_12_ClIN_4_, M = 302.55 gmol^-1^, orange colour plate, 0.14 × 0.11 × 0.07 mm^3^, monoclinic, space group *P*2_1_*/c*, a = 5.9606(12), b = 13.646(3) Å, c = 11.989(2) Å, α = 90°, β = 98.56(3)°, γ = 90°, V = 964.3(3) Å^3^, Z = 4, D_calc_ = 2.084 gcm^3^, F(000) = 584, µ = 3.552 mm^-1^, T = 170 K, θ_max_ = 25.249 °, 6432 total reflections, 1474 with Io > 2σ(Io), R_int_ = 0.0661, 1743 data, 109 parameters, 0 restraints, GooF = 1.066, R_1_ = 0.0378 and wR_2_ = 0.0781 [Io > 2σ(Io)], R_1_ = 0.0483 and wR_2_ = 0.0819 (all reflections), 0.934 < d∆ρ < –1.262 eÅ^3^.

5) Crystal data for [HMTA]·[I_2_]_2_ (**5**): CCDC - 2041022, C_6_H_12_I_4_N_4_, M = 647.80 gmol^-1^, orange colour block, 0.17 × 0.13 × 0.13 mm^3^, monoclinic, space group *P*2_1_*/c*, a = 6.0836(12), b = 16.606(3) Å, c = 14.709(3) Å, α = 90°, β = 92.12(3)°, γ = 90°, V = 1484.9(5) Å^3^, Z = 4, D_calc_ = 2.898 gcm^3^, F(000) = 1152, µ = 8.371 mm^-1^, T = 170 K, θ_max_ = 25.248 °, 9999 total reflections, 2297 with Io > 2σ(Io), R_int_ = 0.0404, 2692 data, 127 parameters, 0 restraints, GooF = 1.044, R_1_ = 0.0305 and wR_2_ = 0.0548 [Io > 2σ(Io)], R_1_ = 0.0400 and wR_2_ = 0.0574 (all reflections), 0.639 < d∆ρ < –0.768 eÅ^3^.

6) Crystal data for [HMTA]·[ICl]_2_ (**6**): CCDC - 2041023, C_6_H_12_Cl_2_I_2_N_4_, M = 464.90 gmol^-1^, orange colour block, 0.2 × 0.15 × 0.13 mm^3^, monoclinic, space group *P*2_1_*/c*, a = 5.9773(12), b = 15.148(3) Å, c = 14.032(3) Å, α = 90°, β = 91.91(3)°, γ = 90°, V = 1269.9(4) Å^3^, Z = 4, D_calc_ = 2.432 gcm^3^, F(000) = 864, µ = 5.346 mm^-1^, T = 170 K, θ_max_ = 28.853 °, 10708 total reflections, 2833 with Io > 2σ(Io), R_int_ = 0.0385, 3293 data, 127 parameters, 0 restraints, GooF = 1.038, R_1_ = 0.0278 and wR_2_ = 0.0513 [Io > 2σ(Io)], R_1_ = 0.0354 and wR_2_ = 0.0533 (all reflections), 0.548 < d∆ρ < –0.759 eÅ^3^.

7) Crystal data for [HMTA]·[NBS]_2_ (**7**): CCDC - 2041024, C_29_H_41_Br_4_Cl_3_N_12_O_8_, M = 1111.73 gmol^-1^, colourless block, 0.11 × 0.09 × 0.08 mm^3^, triclinic, space group *P*-1, a = 6.7019(13), b = 7.1733(14) Å, c = 21.591(4) Å, α = 98.26(3) °, β = 96.45(3)°, γ = 99.19(3) °, V = 1004.3(4) Å^3^, Z = 1, D_calc_ = 1.838 gcm^3^, F(000) = 554, µ = 4.271 mm^-1^, T = 170 K, θ_max_ = 25.249 °, 6946 total reflections, 3041 with Io > 2σ(Io), R_int_ = 0.0299, 3602 data, 271 parameters, 0 restraints, GooF = 1.139, R_1_ 0.0417 and wR_2_ = 0.0957 [Io > 2σ(Io)], R_1_ = 0.0522 and wR_2_ = 0.0995 (all reflections), 0.553 < d∆ρ < –0.431 eÅ^3^.

8) Crystal data for [HMTA]·[NBP]_2_ (**8**): CCDC - 2041025, C_11_H_10_BrN_3_O_2_, M = 296.13 gmol^-1^, colourless plate, 0.07 × 0.068 × 0.066 mm^3^, orthorhombic, space group *Pmn2_1_*, a = 31.646(6), b = 5.7998(7) Å, c = 5.9963(9) Å, α = 90°, β = 90°, γ = 90°, V = 1100.5(3) Å^3^, Z = 4, D_calc_ = 1.787 gcm^3^, F(000) = 592, µ = 3.728 mm^-1^, T = 120 K, θ_max_ = 30.455°, 16153 total reflections, 2137 with Io > 2σ(Io), R_int_ = 0.1428, 3206 data, 156 parameters, 1 restraints, Flack parameter x = –0.009(15), GooF = 1.010, R_1_ = 0.0700 and wR_2_ = 0.0733 [Io > 2σ(Io)], R_1_ = 0.1227 and wR_2_ = 0.0874 (all reflections), 0.796 < d∆ρ < –0.808 eÅ^3^.

9) Crystal data for [HMTA]·[NBS]_4_ (**9**): CCDC - 2041026, C_42_H_56_Br_7_Cl_4_N_15_O_14_, M = 1696.18 gmol^-1^, colourless block, 0.13 × 0.11 × 0.09 mm^3^, triclinic, space group *P*-1, a = 7.4375(15), b = 12.392(3) Å, c = 17.214(3) Å, α = 102.13(3)°, β = 94.58(3)°, γ = 103.14(3)°, V = 1496.9(6) Å^3^, Z = 2, D_calc_ = 1.882 gcm^3^, F(000) = 838, µ = 4.942 mm^-1^, T = 170 K, θ_max_ = 25.249°, 11370 total reflections, 3115 with Io > 2σ(Io), R_int_ = 0.0792, 5387 data, 391 parameters, 36 restraints, GooF = 1.026, R_1_ = 0.0710 and wR_2_ = 0.1261 [Io > 2σ(Io)], R_1_ = 0.1457 and wR_2_ = 0.1516 (all reflections), 0.789 < d∆ρ < –0.751 eÅ^3^.

10) Crystal data for [HMTA]·[NBS]_4a_ (**10**): CCDC - 2041027, C_24_H_28_Br_4_Cl_10_N_8_O_8_, M = 1230.68 gmol^-1^, colourless block, 0.16 × 0.14 × 0.1 mm^3^, tetragonal, space group *P4_2_/nmc*, a = 17.682(3), b = 17.682(3) Å, c = 7.2014(14) Å, α = 90°, β = 90°, γ = 90°, V = 2251.5(8) Å^3^, Z = 2, D_calc_ = 1.815 gcm^3^, F(000) = 1204, µ = 4.218 mm^-1^, T = 170 K, θ_max_ = 28.878 °, 20226 total reflections, 1157 with Io > 2σ(Io), R_int_ = 0.0647, 1567 data, 105 parameters, 0 restraints, GooF = 1.051, R_1_ = 0.0358 and wR_2_ = 0.0860 [Io > 2σ(Io)], R_1_ = 0.0577 and wR_2_ = 0.0949 (all reflections), 0.601 < d∆ρ < –0.553 eÅ^3^.

11) Crystal data for [HMTA]·[NIS]_2_ (**11**): CCDC - 2041028, C_14_H_20_I_2_N_6_O_4_, M = 590.16 gmol^-1^, colourless plate, 0.189 × 0.127 × 0.041 mm^3^, monoclinic, space group *P*2_1_*/c*, a = 7.3937(3), b = 7.0761(3) Å, c = 18.4375(9) Å, α = 90°, β = 93.580(4)°, γ = 90°, V = 962.74(7) Å^3^, Z = 2, D_calc_ = 2.036 gcm^3^, F(000) = 568, µ = 3.299 mm^-1^, T = 120 K, θ_max_ = 25.249 °, 1737 total reflections, 1424 with Io > 2σ(Io), R_int_ = 0.0444 (for overall data), 1737 data, 116 parameters, 7 restraints, GooF = 1.155, R_1_ = 0.0433 and wR_2_ = 0.0890 [Io > 2σ(Io)], R_1_ = 0.0568 and wR_2_ = 0.0970 (all reflections), 1.341 < d∆ρ < –0.852 eÅ^3^.

12) Crystal data for [HMTA]·[NISac] (**12**): CCDC - 2041029, C_13_H_16_IN_5_O_3_S, M = 449.27 gmol^-1^, colourless plate, 0.13 × 0.09 × 0.05 mm^3^, monoclinic, space group *P*2_1_, a = 7.7514(16) Å, b = 7.6524(15) Å, c = 13.862(3) Å, α = 90°, β = 97.87(3)°, γ = 90°, V = 814.5(3) Å^3^, Z = 2, D_calc_ = 1.832 gcm^3^, F(000) = 444, µ = 2.116 mm^-1^, T = 170 K, θ_max_ = 25.249 °, 5916 total reflections, 1481 with Io > 2σ(Io), R_int_ = 0.1124, 2811 data, 196 parameters, 7 restraints, Flack parameter x = 0.27(6), GooF = 1.131, R_1_ = 0.0935 and wR_2_ = 0.1056 [Io > 2σ(Io)], R_1_ = 0.1972 and wR_2_ = 0.1330 (all reflections), 0.950 < d∆ρ < –1.106 eÅ^3^.

13) Crystal data for [bis(HMTA)I]I_3_ (**13**): CCDC - 2041030, C_12_H_24_I_4_N_8_, M = 787.99 gmol^-1^, brown block, 0.13 × 0.09 × 0.05 mm^3^, monoclinic, space group *P*2_1_, a = 5.9621(12) Å, b = 18.506(4) Å, c = 9.785(2) Å, α = 90°, β = 103.26(3)°, γ = 90°, V = 1050.8(4) Å^3^, Z = 2, D_calc_ = 2.490 gcm^3^, F(000) = 728, µ = 5.944 mm^-1^, T = 170 K, θ_max_ = 25.243°, 7953 total reflections, 3031 with Io > 2σ(Io), R_int_ = 0.0556, 3705 data, 187 parameters, 1 restraints, Flack parameter x = 0.41(6), GooF = 1.077, R_1_ = 0.0494 and wR_2_ = 0.0827 [Io > 2σ(Io)], R_1_ = 0.0697 and wR_2_ = 0.0896 (all reflections), 0.920 < d∆ρ < -0.844 eÅ^3^.

14) Crystal data for [HMTA]·[Ipfb]_2_: CCDC - 2041031, C_18_H_12_F_10_I_2_N_4_, M = 728.12 gmol^-1^, colourless block, 0.163× 0.136× 0.116 mm^3^, monoclinic, space group *P*2_1_*/c*, a = 5.97390(10) Å, b = 14.6245(4) Å, c = 24.7926(6) Å, α = 90°, β = 90.905(2)°, γ = 90°, V = 2165.74(9) Å^3^, Z = 4, D_calc_ = 2.233 gcm^3^, F(000) = 1376, µ = 3.005 mm^-1^, T = 170 K, θ_max_ = 25.244°, 14770 total reflections, 3443 with Io > 2σ(Io), R_int_ = 0.0329, 3912 data, 307 parameters, 0 restraints, GooF = 1.041, R_1_ = 0.0227 and wR_2_ = 0.0435 [Io > 2σ(Io)], R_1_ = 0.0278 and wR_2_ = 0.0459 (all reflections), 0.385 < d∆ρ < –0.377 eÅ^3^.

15) Crystal data for [HMTA]·[Ditfb]_2_: CCDC - 2041032, C_12_H_12_F_4_I_2_N_4_, M = 542.06 gmol^-1^, colourless block, 0.208 × 0.161 × 0.108 mm^3^, monoclinic, space group *P*2_1_*/m*, a = 5.8965(5) Å, b = 21.2497(14) Å, c = 6.3175(5) Å, α = 90°, β = 91.866(7)°, γ = 90°, V = 791.16(11) Å^3^, Z = 2, D_calc_ = 2.275 gcm^3^, F(000) = 508, µ = 4.017 mm^-1^, T = 170 K, θ_max_ = 25.247°, 5017 total reflections, 1329 with Io > 2σ(Io), R_int_ = 0.0304, 1474 data, 106 parameters, 0 restraints, GooF = 1.051, R_1_ = 0.0201 and wR_2_ = 0.0429 [Io > 2σ(Io)], R_1_ = 0.0246 and wR_2_ = 0.0465 (all reflections), 0.883 < d∆ρ < –0.617 eÅ^3^.

## 4. X-Ray crystal structures


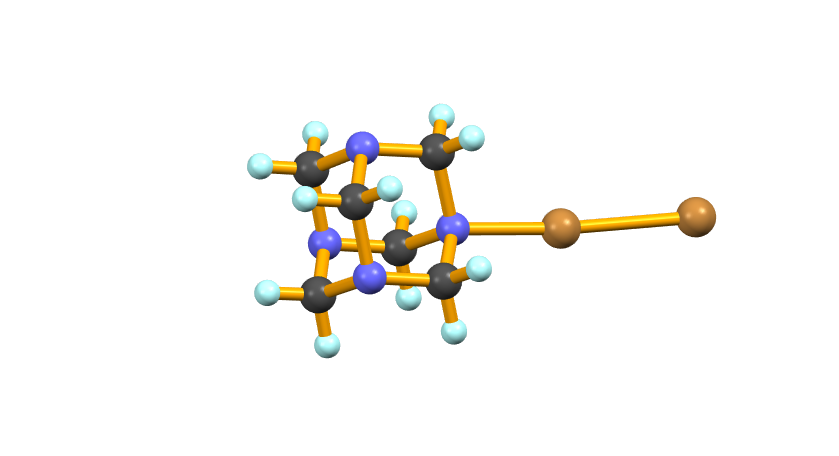


**Supplementary Figure 1**. X-Ray crystal structure of [HMTA]·[Br_2_] (**1**) in ball and stick model.


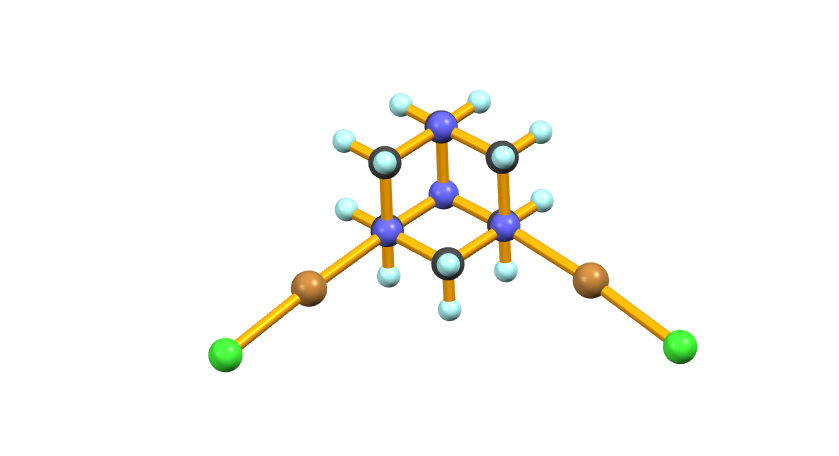


**Supplementary Figure 2**. X-Ray crystal structure of [HMTA]·[BrCl]_2_ (**2**) in ball and stick model.


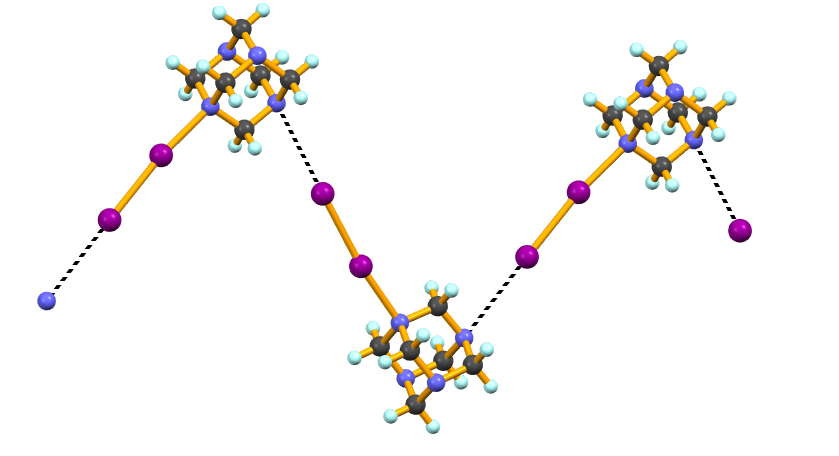


**Supplementary Figure 3**. 1D polymeric view of [HMTA]·[I_2_] (**3**) in ball and stick model. Black dotted lines represent halogen bonding at distances of 3.519 Å.


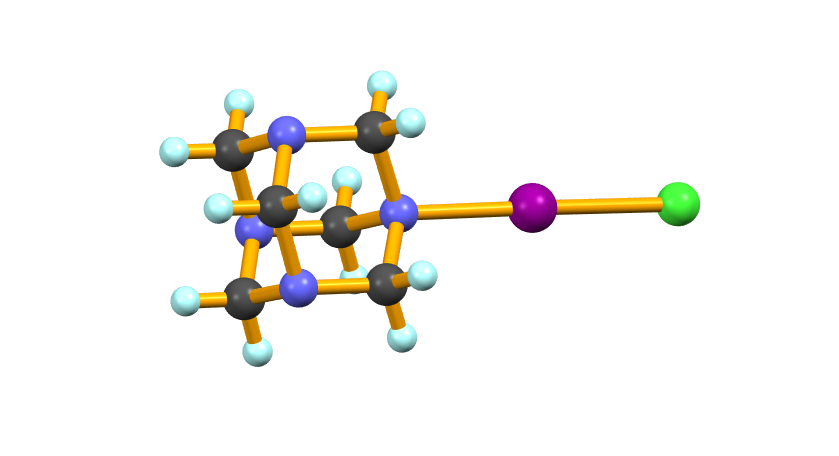


**Supplementary Figure 4**. X-Ray crystal structure of [HMTA]·[ICl] (**4**) in ball and stick model.


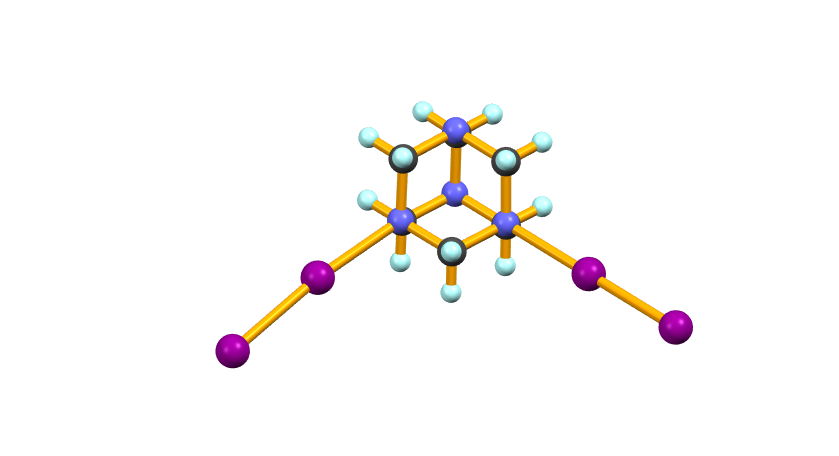


**Supplementary Figure 5**. X-Ray crystal structure of [HMTA]·[I_2_]_2_ (**5**) in ball and stick model.


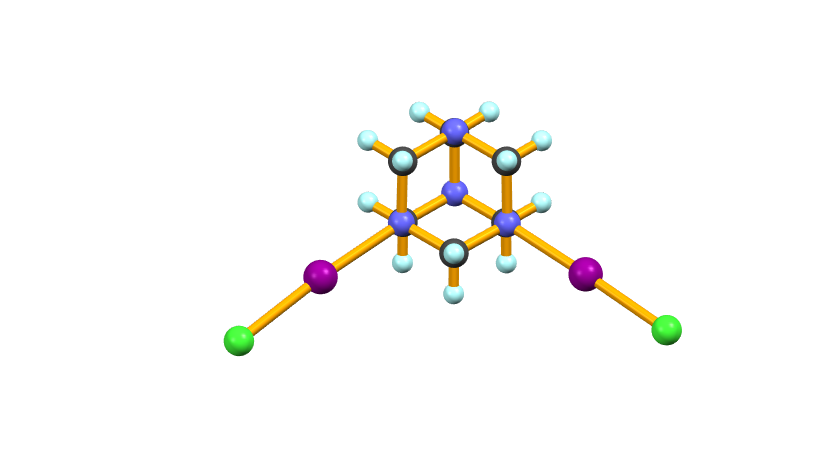


**Supplementary Figure 6**. X-Ray crystal structure of [HMTA]·[ICl]_2_ (**6**) in ball and stick model.


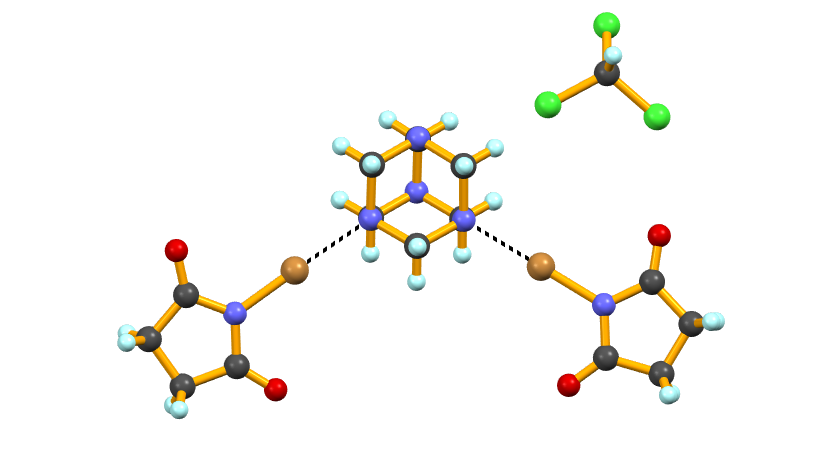


**Supplementary Figure 7**. X-Ray crystal structure of [HMTA]·[NBS]_2_ (**7**) in ball and stick model. Black dotted lines represent halogen bonding interactions. The structure co-crystallizes with CHCl_3_ molecule.


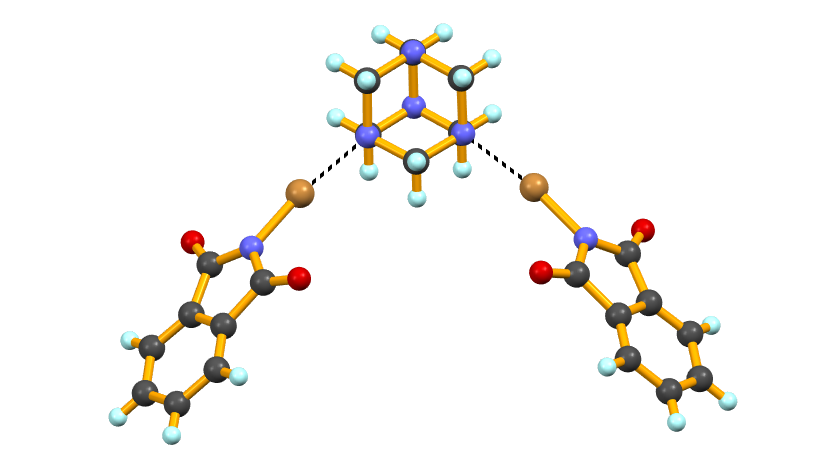


**Supplementary Figure 8**. X-Ray crystal structure of [HMTA]·[NBP]_2_ (**8**) in ball and stick model. Black dotted lines represent halogen bonding interactions.


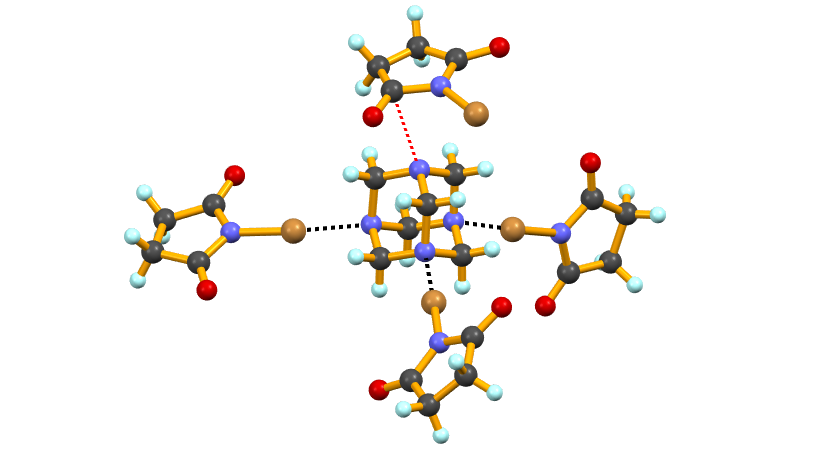


**Supplementary Figure 9**. X-Ray crystal structure of [HMTA]·[NBS]_4_ (**9**) in ball and stick model. Black dotted lines represent halogen bonding interactions and red are N･･･C interactions. Note: subscript 4 represents tridentate halogen bond coordination mode of HMTA. Out of four crystallographically independent NBS donors one NBS do not participate in the halogen bonding.


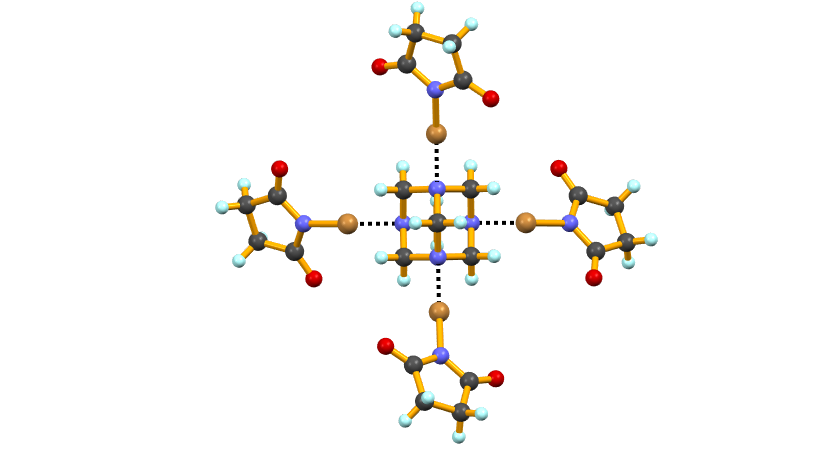


**Supplementary Figure 10**. X-Ray crystal structure of [HMTA]·[NBS]_4a_ (**10**) in ball and stick model. Black dotted lines represent halogen bonding interactions. Note: [HMTA]·[NBS]_4a,_ subscript 4a represents tetradentate halogen bonding mode of HMTA.


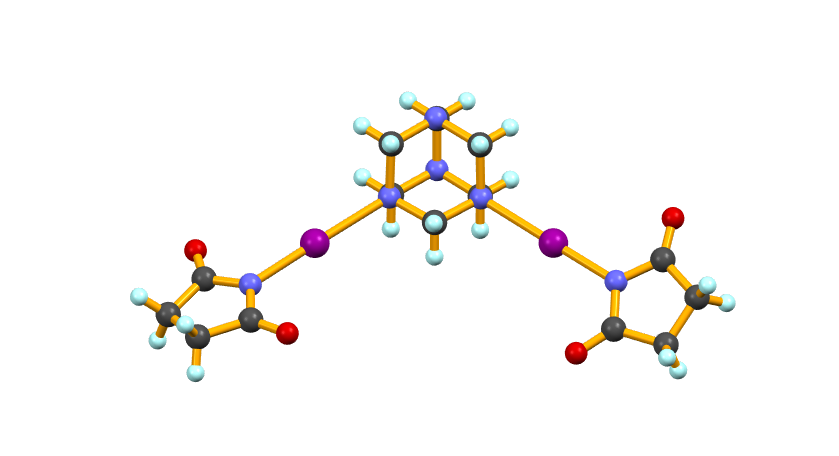


**Supplementary Figure 11**. X-Ray crystal structure of [HMTA]·[NIS]_2_ (**11**) in ball and stick model.


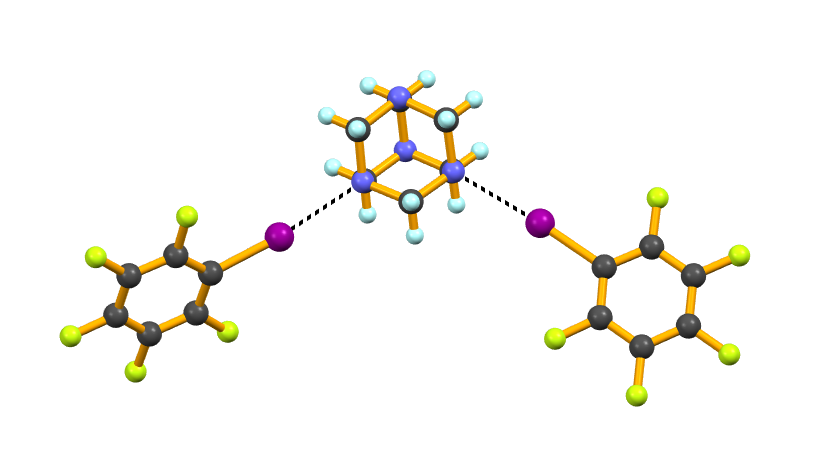


**Supplementary Figure 12.** X-Ray crystal structure of [HMTA]·[Ipfb]_2_ in ball and stick model. Black dotted lines represent halogen bonding interactions.


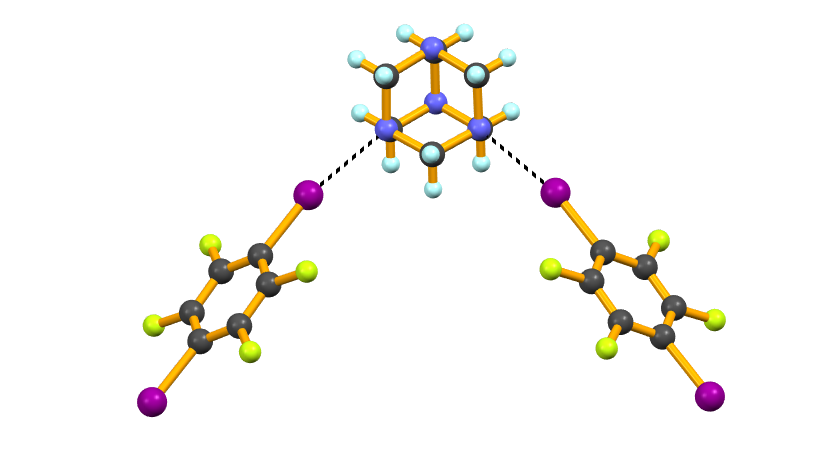


**Supplementary Figure 13.** X-Ray crystal structure of [HMTA]·[Ditfb]_2_ in ball and stick model. Black dotted lines represent halogen bonding interactions.


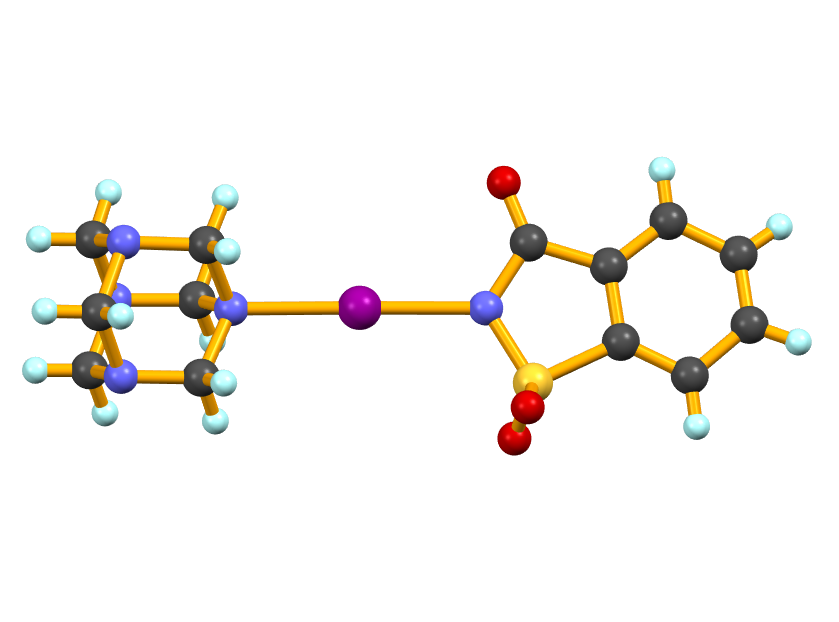


**Supplementary Figure 14.** X-Ray crystal structure of [HMTA]·[NISac] (**12**) in ball and stick model.


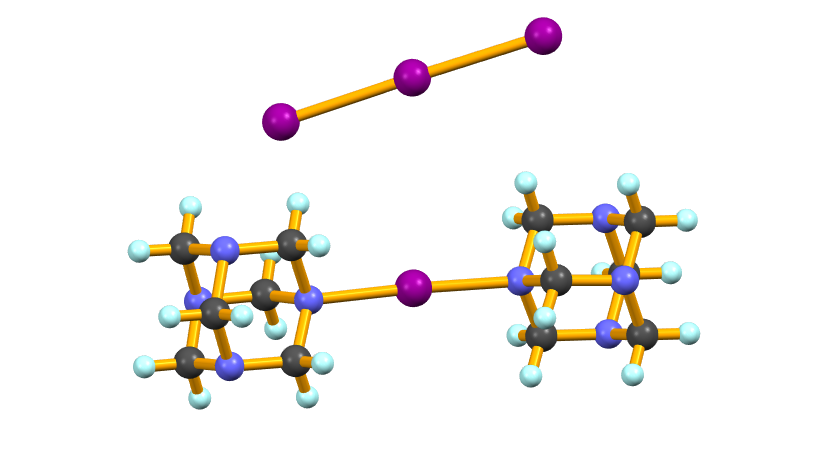


**Supplementary Figure 15.** X-Ray crystal structure of [bis(HMTA)I]I_3_ (**13**) in ball and stick model.

**5. The Cambridge Crystallographic Database Search**

The X-ray crystal structure bond parameters of halogen bonds were obtained by using the Cambridge Crystallographic Database (CSD). Critical evaluation of literature data on halogen bonding analysis was not attempted. Conquest program (Version 2020.2.0) is used to search to all type halogen bonds. Note, some values and esd errors may be uncorrected and listed as reported.

**Table S4**: Halogen bonding parameters of [HMTA][NBS]_n_.

| **Entry** | **Refcode** | ***d*(N–Br) [Å]** | ***d*(B**･･･**N) [Å]** | **∠(N–Br**･･･**N) [°]** | ***d*(N･･･N′) [Å]** |
| --- | --- | --- | --- | --- | --- |
| 1 | IBIYUP | 1.917(3)  1.925(3) | 2.433(3)  2.414(3) | 172.04(12)  173.63(11) | 4.339(4)  4.332(5) |

**Table S5**: Halogen bonding parameters of [HMTA][Br_2_]_n_.

| **Entry** | **Refcode** | **donor** | ***d*(B**･･･**O) [Å]** | **∠(N–Br**･･･**N) [°]** | ***d*(C･･･N) [Å]** |
| --- | --- | --- | --- | --- | --- |
| 1 | ZZZHEM | 2(Br_2_) | No coordinates | -- | -- |

**Table S6**: Halogen bonding parameters of [HMTA][NIS]_n_.

| **Entry** | **Refcode** | ***d*(**N–I**) [Å** | ***d*(I**･･･N**) [Å]** | **∠(N–I**･･･N**) [°]** | ***d*(N･･･N′) [Å]** |
| --- | --- | --- | --- | --- | --- |
| 1 | YANGIF | 2.094(2)  2.099(2) | 2.563(2)  2.541(2) | 174.55(10)  176.8(1) | 4.652(3)  4.638(3) |
| 2 | YANGEB | 2.118(3) | 2.542(3) | 175.50(11) | 4.656(4) |
| 3 | YANGAX | 2.096(3)  2.103(3) | 2.516(3)  2.486(3) | 175.20(11)  179.48(12) | 4.609(4)  4.589(4) |
| 4 | YANFUQ | 2.096(3)  2.103(3 | 2.516(3)  2.486(3) | 175.20(11)  179.48(12) | 4.609(4)  4.589(4) |
| 5 | YANFOK01 | 2.099(3)  2.086(3) | 2.523(3)  2.586(3) | 176.72(11)  176.66(12) | 4.620(4)  4.670(4) |
| 6 | YANFOK | 2.101(5) | 2.520(5) | 179.6(2) | 4.622(8) |
| 7 | IBIZAW | 2.121(9) | 2.549(8) | 175.2(3) | 4.666(11) |

**Table S7**: Halogen bonding parameters of [HMTA][I_2_]_n_.

| **Entry** | **CSD-Refcode** | **donor** | ***d*(I**･･･N**) [Å]** | **∠(I–I**･･･N**) [°]** | ***d*(I･･･N′) [Å]** |
| --- | --- | --- | --- | --- | --- |
| 1 | HMTITI | I⁺ | 2.2999(4)  2.3055(4) | 176.5168(7) | 4.6032(7) |
| 2 | HMTNTI | (NI_3_)I_2_^b^ | 2.5829(3)  2.5661(2) | 175.3816(4)  178.5972(8) | 4.7242(5)  4.7058(4) |
| 3 | HXMIOD | 2(I2) | 2.4971(3)  2.4980(4) | 173.9194(9)  173.8536(11) | 5.2805(7)  5.2615(9) |
| 4 | HXMTDI | I_2_ | 2.4389(4) | 173.0742(4) | 5.2589(9) |
| 5 | YUYNUB | 3(I_2_) | 2.593(6) | 177.58(16) | 5.338(6) |

^a^Entry 2 is not considered for bond distance comparisons due to the presence of NI_3_ species.

**Table S8**: Halogen bonding parameters of pyridine-I_2_ complexes^a, b^.

| **Entry** | **CSD-Refcode** | **acceptor** | ***d*(I･･･N) [Å]** | ***d*(I–I) [Å]** | **∠(I–I･･･N) [°]** |
| --- | --- | --- | --- | --- | --- |
| 1 | VUHDIN | Py | 2.425(8) | 2.8043(9 | 176.44(18) |
| 2 | LEWKOP | Acety-Py | 2.358(6) | -- | 176.08(17) |
| 3 | PEWQIT | Opvp | 2.352(4) | 2.8298(7) | 177.19(11) |

^a^Entries 2 and 3 are not considered for bond distance comparisons due to the aggregation effect on the halogen bonding. ^b^Abbreviations: Pyridine = Py; Acetylpyridine = Acety-Py; 4-(2-(4-(octyloxy)phenyl)vinyl)pyridine = Opvp.

**Table S9**: Halogen bonding parameters of pyridine-IBr complexes^a,b^.

| **Entry** | **CSD-Refcode** | **acceptor** | ***d*(I･･･N) [Å]** | ***d*(I–Br) [Å]** | **∠(Br–I･･･N) [°]** |
| --- | --- | --- | --- | --- | --- |
| 1 | PYIOBR04 | Py | 2.313(5) | 2.6631(7) | 178.75(12) |
| 2 | PYIOBR03 | Py | 2.304(3) | 2.6535(4) | 178.57(7) |
| 3 | PYIOBR02 | Py | 2.318(4) | 2.649(5) | 178.28(19) |
| 4 | PYIOBR01 | Py | 2.323(5) | 2.6385(9) | 179.20(11) |
| 5 | PYIOBR | Py | Coordinates  not available | -- | -- |
| 6 | TAGNIZ | Py | 2.461(13) | 2.577(3) | 175.8(3) |
| 7 | TASPEJ | Pz | 2.405(3)  2.409(3) | 2.5952(4)  2.5879(5) | 173.99(6)  175.14(6) |

^a^Entry 7 is not considered for bond distance comparison due to its polydentate nature and other additional electronic effects on the halogen bonding. ^b^Abbreviations: Pyridine = Py; Pyrazine = Pz.

**Table S10**: Halogen bonding parameters of pyridine-ICl complexes^a^.

| **Entry** | **CSD-Refcode** | **acceptor** | ***d*(I･･･N) [Å]** | ***d*(I–Cl) [Å]** | **∠(Cl–I･･･N) [°]** |
| --- | --- | --- | --- | --- | --- |
| 1 | PYRIICl2 | Py | 2.281(4) | 2.5042(16) | 179.41(12) |
| 2 | PYRIICl1 | Py | 2.284(3) | 2.5232(10) | 179.18(9) |
| 3 | PYRIICl10 | Py | 2.2901(5) | 2.5098(5) | 178.72(2) |
| 4 | ZUDSEZ | 2BrPy | 2.351(12) | 2.476(4) | 177.6(3) |
| 5 | GANXAV01 | 3BrPy | 2.330(5) | 2.4665(16) | 178.49(13) |
| 6 | GANXAV | 3BrPy | 2.3437(18) | 2.4734(7) | 178.43(5) |
| 7 | ZUDSID | 3BrPy | 2.30(2) | 2.489(6) | 176.8(6) |
| 8 | GANXOJ | 4DMAP | 2.2462(19) | 2.5615(7) | 179.24(5) |
| 9 | GANXID | 246MePy | 2.294(5) | 2.5312(16) | 179.05(14) |
| 10 | YOFKEM | 4NH_2_Py | 2.186(5) | 2.6337(13) | 178.38(13) |
| 11 | TAGNEV01 | 2,2BiPy | 2.3214(18)  2.337(2) | 2.4974(7)  2.4878(9) | 179.41(5)  175.33(5) |
| 12 | TAGNEV | 2,2BiPy | 2.336(8)  2.343(7) | 2.459(3)  2.478(3) | 179.3(2)  176.2(2) |

^a^Abbreviations: Pyridine = Py; 2-bromopyridine = 2BrPy; 4-(dimethylamino)pyridine = 4DMAP; 2,4,6-trimethylpyridine = 246MePy; 4-aminopyridine = 4NH_2_Py; 2,2’-bipyridine = 2,2BiPy.

## Computational studies

The energies of the noncovalent complexes included in this study were computed using the RI-MP2/def2-TZVP level of theory (Handy and Schaefer, 1984; Weigend and Ahlrichs, 2005) by means of the program TURBOMOLE version 7.0 (Ahlrichs et al., 1989). For the additivity study we have fully optimized the structures. The interaction energies were calculated with correction for the basis set superposition error (BSSE) by using the Boys–Bernardi counterpoise technique (Boys and Bernardi, 1970). To evaluate the interactions in the solid state, we have used the crystallographic coordinates at the RI-MP2/def2-TZVP level of theory. The Bader’s "Atoms in molecules" theory (Bader, 1991) has been used to characterize the interactions discussed herein by means of the AIMall calculation package (Version 13.05.06), Todd A. Keith, TK Gristmill Software, Overland Park KS, USA, 2013).


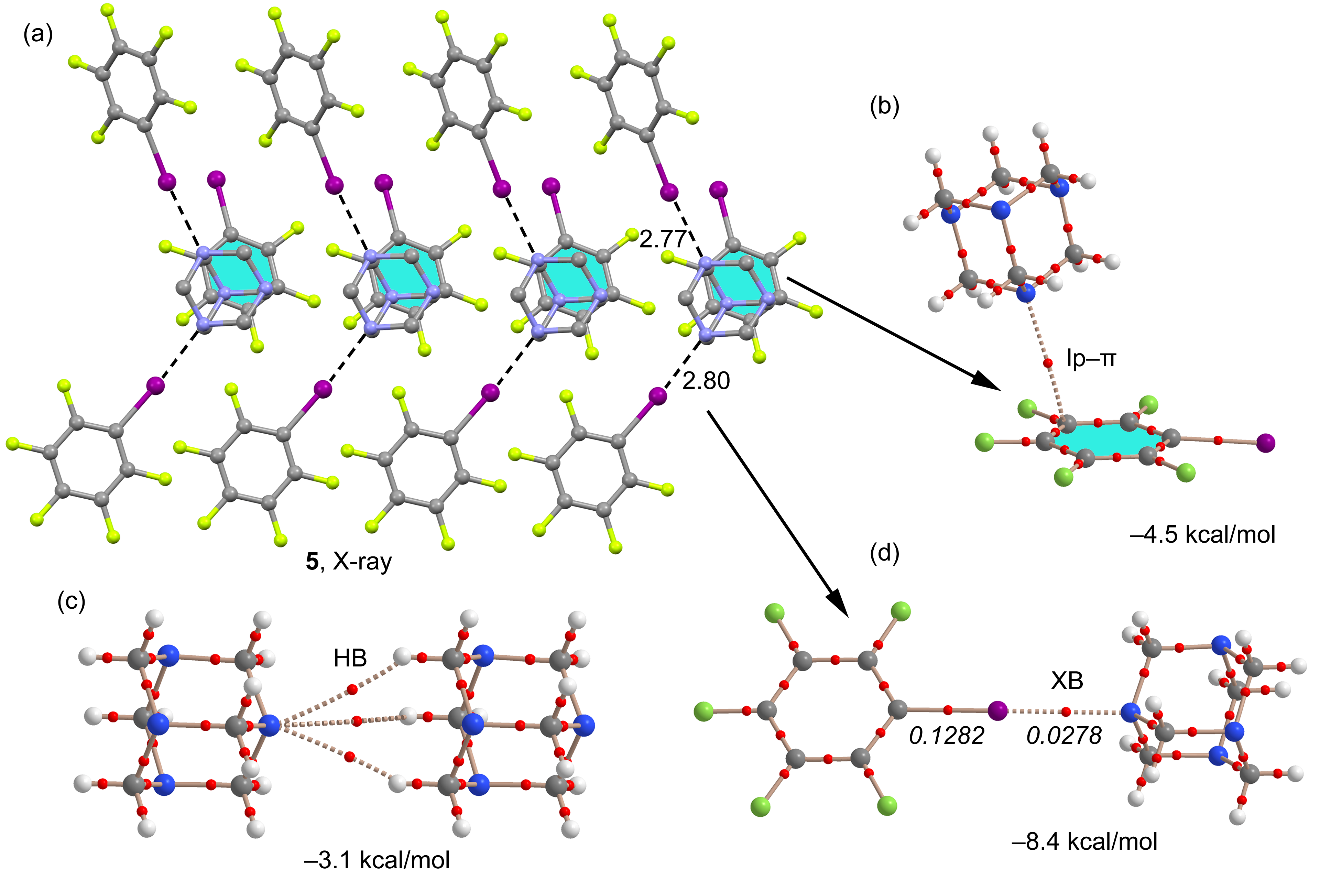


**Supplementary Figure 16.** (a) Partial packing view of [HMTA]·[Ipfb]_2_. Theoretical dimeric models and AIM analysis displaying (b) lone-pair, (c) C–H···N, and (d) C–I···N interactions. Bond critical points are red dots. Interaction energies in kcal/mol and distances in Å. The values in italics correspond to the ρ(r) values at the bond critical points in a.u.

**References**

Ahlrichs, R., Bär, M., Häser, M., Horn, H., and Kölmel, C. (1989). Electronic structure calculations on workstation computers: The program system turbomole. *Chem. Phys. Lett.* 162, 165–169. doi:https://doi.org/10.1016/0009-2614(89)85118-8.

Bader, R. F. W. (1991). A quantum theory of molecular structure and its applications. *Chem. Rev.* 91, 893–928. doi:10.1021/cr00005a013.

Boys, S. F., and Bernardi, F. (1970). The calculation of small molecular interactions by the differences of separate total energies. Some procedures with reduced errors. *Mol. Phys.* 19, 553–566. doi:10.1080/00268977000101561.

Dolomanov, O. V., Bourhis, L. J., Gildea, R. J., Howard, J. A. K., and Puschmann, H. (2009). *OLEX2*: A complete structure solution, refinement and analysis program. *J. Appl. Crystallogr.* 42, 339–341. doi:10.1107/S0021889808042726.

Handy, N. C., and Schaefer, H. F. (1984). On the evaluation of analytic energy derivatives for correlated wave functions. *J. Chem. Phys.* 81, 5031–5033. doi:10.1063/1.447489.

Hooft R. W. W. (1998). COLLECT. Nonius BV, Delft, The Netherlands.

Otwinowski, Z., and Minor, W. (1997). “Processing of X-ray diffraction data collected in oscillation mode,” in *Methods in Enzymology*, ed. J. B. T.-M. in E. Charles W. Carter (Academic Press), 307–326. doi:10.1016/S0076-6879(97)76066-X.

Rigaku (2017). CrysAlisPro Software system. *Rigaku Oxford Diffr. 2017., Version 38.46, Rigaku Corportation, Oxford, UK.*

Sheldrick, G. M. (1996). SADABS Version 2008/2. University of Göttingen, Germany.

Sheldrick, G. M. (2008). A short history of SHELX. *Acta Crystallogr. Sect. A Found. Crystallogr.* 64, 112–122. doi:10.1107/S0108767307043930.

Sheldrick, G. M. (2015). Crystal structure refinement with SHELXL. *Acta Crystallogr. Sect. C Struct. Chem.* 71, 3–8. doi:10.1107/S2053229614024218.

Weigend, F., and Ahlrichs, R. (2005). Balanced basis sets of split valence, triple zeta valence and quadruple zeta valence quality for H to Rn: Design and assessment of accuracy. *Phys. Chem. Chem. Phys.* 7, 3297–3305. doi:10.1039/B508541A.
