# Supplementary material for: Short X···N Halogen Bonds With Hexamethylenetetraamine as the Acceptor
Source: Front Chem. 2021 Apr 29;9:623595. doi: 10.3389/fchem.2021.623595 (PMC8116742; doi:10.3389/fchem.2021.623595)

checkCIF/PLATON report

Structure factors have been supplied for datablock(s) HMTA-NBS_4a

THIS REPORT IS FOR GUIDANCE ONLY. IF USED AS PART OF A REVIEW PROCEDURE FOR PUBLICATION, IT SHOULD NOT REPLACE THE EXPERTISE OF AN EXPERIENCED CRYSTALLOGRAPHIC REFEREE.

No syntax errors found.   CIF dictionary   Interpreting this report

Datablock: HMTA-NBS_4a

```
Bond precision:  C-C = 0.0070 A          Wavelength=0.71073

Cell:              a=17.682(3)      b=17.682(3)      c=7.2014(14)
                   alpha=90         beta=90          gamma=90
Temperature:       170 K
```

	Calculated	Reported
Volume	2251.5(9)	2251.5(8)
Space group	P 42/n m c	P 42/n m c
Hall group	-P 4ac 2a	-P 4ac 2a
Moiety formula	2(C Cl4), C6 H12 N4, 4(C4 H4 Br N O2), 4(Cl0.50)	2(C8 H8 Br2 N2 O4), C2 Cl10, C6 H12 N4
Sum formula	C24 H28 Br4 Cl10 N8 O8	C24 H28 Br4 Cl10 N8 O8
Mr	1230.64	1230.68
Dx,g cm-3	1.815	1.815
Z	2	2
Mu (mm-1)	4.219	4.218
F000	1204.0	1204.0
F000’	1205.11	
h,k,lmax	24,24,9	24,24,9
Nref	1569	1567
Tmin,Tmax	0.514,0.656	0.567,0.746
Tmin’	0.504	

```
Correction method= # Reported T Limits: Tmin=0.567 Tmax=0.746
AbsCorr = MULTI-SCAN

Data completeness= 0.999          Theta(max)= 28.878

R(reflections)= 0.0358( 1157)     wR2(reflections)= 0.0949( 1567)

S = 1.051                   Npar= 105
```

The following ALERTS were generated. Each ALERT has the format
test-name_ALERT_alert-type_alert-level.
Click on the hyperlinks for more details of the test.

Alert level A

```
PLAT431_ALERT_2_A Short Inter HL..A Contact  Br1      ..N1       .       2.40 Ang.
                                                      x,y,z  =      1_555 Check
```

Author Response: Halogen bonding

Alert level C

```
PLAT215_ALERT_3_C Disordered Cl1       has ADP max/min Ratio .....        3.4 Note
PLAT241_ALERT_2_C High   'MainMol' Ueq as Compared to Neighbors of         C5 Check
PLAT250_ALERT_2_C Large U3/U1 Ratio for Average U(i,j) Tensor ....        2.6 Note
PLAT260_ALERT_2_C Large Average Ueq of Residue Including       Cl1      0.118 Check
PLAT329_ALERT_4_C Carbon Atom Hybridisation Unclear for ..........         C4 Check
PLAT329_ALERT_4_C Carbon Atom Hybridisation Unclear for ..........         C5 Check
PLAT336_ALERT_2_C Long Bond Distance for ..... C7_a     -Cl1            1.919 Ang.
PLAT341_ALERT_3_C Low Bond Precision on  C-C Bonds ...............      0.007 Ang.
PLAT906_ALERT_3_C Large K Value in the Analysis of Variance ......      3.008 Check
```

Alert level G

```
PLAT004_ALERT_5_G Polymeric Structure Found with Maximum Dimension          1 Info
PLAT012_ALERT_1_G No       _shelx_res_checksum Found in CIF ......     Please Check
PLAT042_ALERT_1_G Calc. and Reported MoietyFormula Strings  Differ     Please Check
PLAT300_ALERT_4_G Atom Site Occupancy of Cl1        Constrained at        0.5 Check
PLAT300_ALERT_4_G Atom Site Occupancy of Cl3        Constrained at       0.25 Check
PLAT300_ALERT_4_G Atom Site Occupancy of Cl4        Constrained at       0.25 Check
PLAT300_ALERT_4_G Atom Site Occupancy of C7         Constrained at       0.25 Check
PLAT300_ALERT_4_G Atom Site Occupancy of H1A        Constrained at        0.5 Check
PLAT300_ALERT_4_G Atom Site Occupancy of H1B        Constrained at        0.5 Check
PLAT300_ALERT_4_G Atom Site Occupancy of H2A        Constrained at        0.5 Check
PLAT300_ALERT_4_G Atom Site Occupancy of H2B        Constrained at        0.5 Check
PLAT300_ALERT_4_G Atom Site Occupancy of H4A        Constrained at        0.5 Check
PLAT300_ALERT_4_G Atom Site Occupancy of H4B        Constrained at        0.5 Check
PLAT300_ALERT_4_G Atom Site Occupancy of H5A        Constrained at        0.5 Check
PLAT300_ALERT_4_G Atom Site Occupancy of H5B        Constrained at        0.5 Check
PLAT300_ALERT_4_G Atom Site Occupancy of Cl2        Constrained at        0.5 Check
PLAT301_ALERT_3_G Main Residue  Disorder ..............(Resd  1  )       100% Note
PLAT302_ALERT_4_G Anion/Solvent/Minor-Residue Disorder (Resd  4  )       100% Note
PLAT367_ALERT_2_G Long?  C(sp?)-C(sp?) Bond  C3       - C4       .       1.50 Ang.
PLAT367_ALERT_2_G Long?  C(sp?)-C(sp?) Bond  C4       - C5       .       1.50 Ang.
PLAT367_ALERT_2_G Long?  C(sp?)-C(sp?) Bond  C5       - C6       .       1.52 Ang.
PLAT432_ALERT_2_G Short Inter X...Y Contact  Br1      ..C2               3.21 Ang.
                                                      x,y,z  =      1_555 Check
PLAT432_ALERT_2_G Short Inter X...Y Contact  Br1      ..C1               3.22 Ang.
                                                      x,y,z  =      1_555 Check
PLAT432_ALERT_2_G Short Inter X...Y Contact  Br1      ..C1               3.22 Ang.
                                           1-y,1/2+x,-1/2-z  =     12_665 Check
PLAT720_ALERT_4_G Number of Unusual/Non-Standard Labels ..........          1 Note
PLAT764_ALERT_4_G Overcomplete CIF Bond List Detected (Rep/Expd) .       1.17 Ratio
PLAT779_ALERT_4_G Suspect or Irrelevant (Bond) Angle(s) in CIF . #          3 Check
              CL1  -CL4  -C7      7.455   1.555   7.455             40.60 Deg.
PLAT779_ALERT_4_G Suspect or Irrelevant (Bond) Angle(s) in CIF . #         42 Check
              CL4  -C7   -CL1     1.555   1.555   7.455             22.80 Deg.
PLAT779_ALERT_4_G Suspect or Irrelevant (Bond) Angle(s) in CIF . #         45 Check
```

```
             CL1  -C7   -CL4     1.555   1.555   7.455             16.20 Deg.
PLAT779_ALERT_4_G Suspect or Irrelevant (Bond) Angle(s) in CIF . #        54 Check
             CL3  -C7   -CL1     1.555   1.555   9.566             14.70 Deg.
PLAT789_ALERT_4_G Atoms with Negative _atom_site_disorder_group  #         1 Check
PLAT910_ALERT_3_G Missing # of FCF Reflection(s) Below Theta(Min).         2 Note
PLAT912_ALERT_4_G Missing # of FCF Reflections Above STh/L=  0.600         1 Note
PLAT933_ALERT_2_G Number of OMIT Records in Embedded .res File ...         2 Note
PLAT978_ALERT_2_G Number C-C Bonds with Positive Residual Density.         0 Info
```

```
   1 ALERT level A = Most likely a serious problem - resolve or explain
   0 ALERT level B = A potentially serious problem, consider carefully
   9 ALERT level C = Check. Ensure it is not caused by an omission or oversight
  35 ALERT level G = General information/check it is not something unexpected

   2 ALERT type 1 CIF construction/syntax error, inconsistent or missing data
  13 ALERT type 2 Indicator that the structure model may be wrong or deficient
   5 ALERT type 3 Indicator that the structure quality may be low
  24 ALERT type 4 Improvement, methodology, query or suggestion
   1 ALERT type 5 Informative message, check
```

It is advisable to attempt to resolve as many as possible of the alerts in all categories. Often the minor alerts point to easily fixed oversights, errors and omissions in your CIF or refinement strategy, so attention to these fine details can be worthwhile. In order to resolve some of the more serious problems it may be necessary to carry out additional measurements or structure refinements. However, the purpose of your study may justify the reported deviations and the more serious of these should normally be commented upon in the discussion or experimental section of a paper or in the "special_details" fields of the CIF. checkCIF was carefully designed to identify outliers and unusual parameters, but every test has its limitations and alerts that are not important in a particular case may appear. Conversely, the absence of alerts does not guarantee there are no aspects of the results needing attention. It is up to the individual to critically assess their own results and, if necessary, seek expert advice.

Publication of your CIF in IUCr journals

A basic structural check has been run on your CIF. These basic checks will be run on all CIFs submitted for publication in IUCr journals (*Acta Crystallographica*, *Journal of Applied Crystallography*, *Journal of Synchrotron Radiation*); however, if you intend to submit to *Acta Crystallographica Section C* or *E* or *IUCrData*, you should make sure that full publication checks are run on the final version of your CIF prior to submission.

Publication of your CIF in other journals

Please refer to the *Notes for Authors* of the relevant journal for any special instructions relating to CIF submission.

PLATON version of 18/09/2020; check.def file version of 20/08/2020

Datablock HMTA-NBS_4a - ellipsoid plot

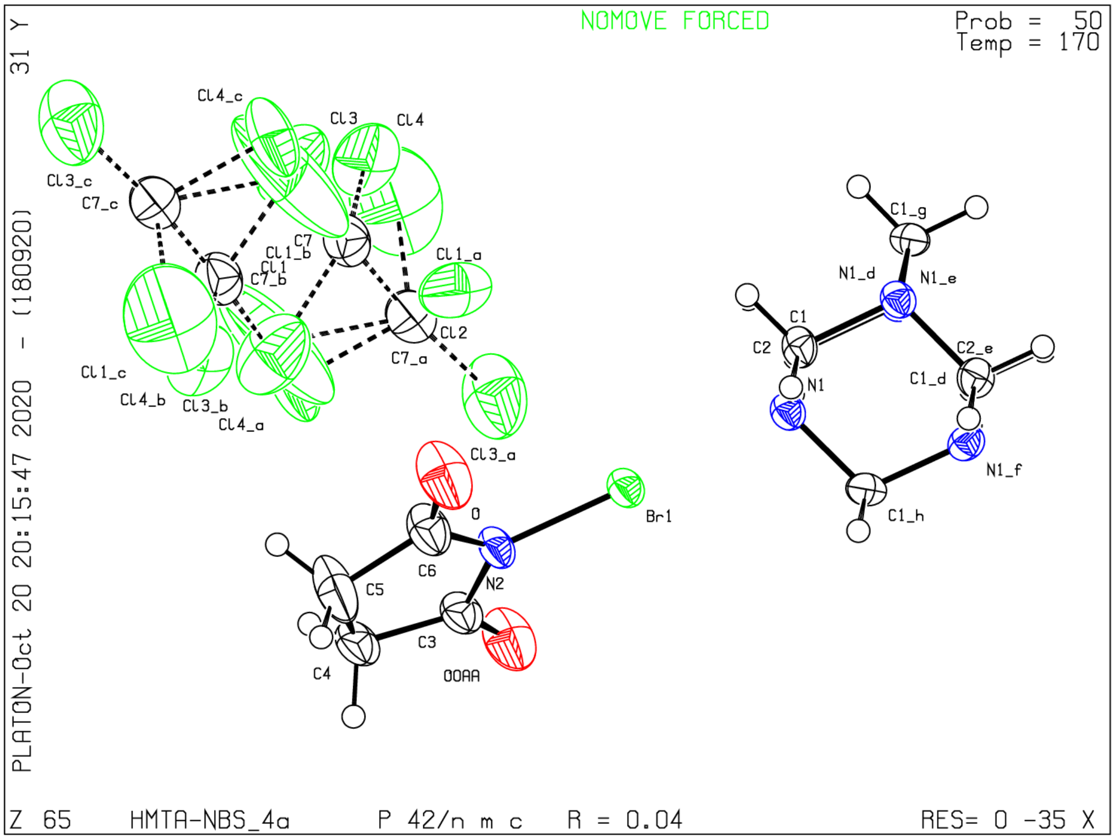

Supplement: Supplementary file 2 [file Data_Sheet_2.ZIP › CIF_MS 623595/[HMTA][NBS]4a_checkcif.pdf]
