# Supplementary material for: Short X···N Halogen Bonds With Hexamethylenetetraamine as the Acceptor
Source: Front Chem. 2021 Apr 29;9:623595. doi: 10.3389/fchem.2021.623595 (PMC8116742; doi:10.3389/fchem.2021.623595)

checkCIF/PLATON report

Structure factors have been supplied for datablock(s) HMTA-NBS_4

THIS REPORT IS FOR GUIDANCE ONLY. IF USED AS PART OF A REVIEW PROCEDURE FOR PUBLICATION, IT SHOULD NOT REPLACE THE EXPERTISE OF AN EXPERIENCED CRYSTALLOGRAPHIC REFEREE.

No syntax errors found.   CIF dictionary   Interpreting this report

Datablock: HMTA-NBS_4

Bond precision: C-C = 0.0144 A   Wavelength=0.71073

Cell:	a=7.4375(15)	b=12.392(3)	c=17.214(3)
	alpha=102.13(3)	beta=94.58(3)	gamma=103.14(3)
Temperature:	170 K		

	Calculated	Reported
Volume	1496.9(6)	1496.9(6)
Space group	P -1	P -1
Hall group	-P 1	-P 1
Moiety formula	2(C6 H12 N4), 7(C4 H4 Br N O2), 2(C H2 Cl2)	7(C4 H4 Br N O2), 2(C H2 Cl2), 2(C6 H12 N4)
Sum formula	C42 H56 Br7 Cl4 N15 O14	C42 H56 Br7 Cl4 N15 O14
Mr	1696.12	1696.18
Dx,g cm-3	1.882	1.882
Z	1	1
Mu (mm-1)	4.942	4.942
F000	838.0	838.0
F000’	836.97	
h,k,lmax	8,14,20	8,14,20
Nref	5417	5387
Tmin,Tmax	0.531,0.641	0.572,0.746
Tmin’	0.521	

Correction method= # Reported T Limits: Tmin=0.572 Tmax=0.746
AbsCorr = MULTI-SCAN

Data completeness= 0.994   Theta(max)= 25.249

R(reflections)= 0.0710(3115)   wR2(reflections)= 0.1516(5387)

S = 1.026   Npar= 391

The following ALERTS were generated. Each ALERT has the format
test-name_ALERT_alert-type_alert-level.
Click on the hyperlinks for more details of the test.

Alert level A

```
PLAT431_ALERT_2_A Short Inter HL..A Contact  Br1      ..N2       .       2.41 Ang.
                                                      x,y,z  =     1_555 Check
```

Author Response: Halogen bonding

```
PLAT431_ALERT_2_A Short Inter HL..A Contact  Br2      ..N1       .       2.38 Ang.
                                                      x,y,z  =     1_555 Check
```

Author Response: Halogen bonding

```
PLAT431_ALERT_2_A Short Inter HL..A Contact  Br3      ..N3       .       2.37 Ang.
                                                      x,y,z  =     1_555 Check
```

Author Response: Halogen bonding

Alert level C

```
PLAT341_ALERT_3_C Low Bond Precision on  C-C Bonds ...............   0.01444 Ang.
PLAT906_ALERT_3_C Large K Value in the Analysis of Variance ......     5.579 Check
PLAT911_ALERT_3_C Missing FCF Refl Between Thmin & STh/L=    0.600        30 Report
PLAT977_ALERT_2_C Check Negative Difference Density on H1SB            -0.32 eA-3
PLAT977_ALERT_2_C Check Negative Difference Density on H5B             -0.32 eA-3
```

Alert level G

```
PLAT003_ALERT_2_G Number of Uiso or Uij Restrained non-H Atoms ...         6 Report
PLAT012_ALERT_1_G No       _shelx_res_checksum Found in CIF ......    Please Check
PLAT042_ALERT_1_G Calc. and Reported MoietyFormula Strings  Differ    Please Check
PLAT083_ALERT_2_G SHELXL Second Parameter in WGHT  Unusually Large      5.87 Why ?
PLAT154_ALERT_1_G The s.u.'s on the Cell Angles are Equal ..(Note)      0.03 Degree
PLAT171_ALERT_4_G The CIF-Embedded .res File Contains EADP Records         1 Report
PLAT186_ALERT_4_G The CIF-Embedded .res File Contains ISOR Records         2 Report
PLAT300_ALERT_4_G Atom Site Occupancy of Br4       Constrained at        0.5 Check
PLAT300_ALERT_4_G Atom Site Occupancy of O7        Constrained at        0.5 Check
PLAT300_ALERT_4_G Atom Site Occupancy of O8        Constrained at        0.5 Check
PLAT300_ALERT_4_G Atom Site Occupancy of N8        Constrained at        0.5 Check
PLAT300_ALERT_4_G Atom Site Occupancy of C19       Constrained at        0.5 Check
PLAT300_ALERT_4_G Atom Site Occupancy of C20       Constrained at        0.5 Check
PLAT300_ALERT_4_G Atom Site Occupancy of C21       Constrained at        0.5 Check
PLAT300_ALERT_4_G Atom Site Occupancy of C22       Constrained at        0.5 Check
PLAT300_ALERT_4_G Atom Site Occupancy of H20A      Constrained at        0.5 Check
PLAT300_ALERT_4_G Atom Site Occupancy of H20B      Constrained at        0.5 Check
PLAT300_ALERT_4_G Atom Site Occupancy of H21A      Constrained at        0.5 Check
PLAT300_ALERT_4_G Atom Site Occupancy of H21B      Constrained at        0.5 Check
PLAT302_ALERT_4_G Anion/Solvent/Minor-Residue Disorder (Resd  5  )      100% Note
PLAT432_ALERT_2_G Short Inter X...Y Contact  Br1      ..C3              3.16 Ang.
                                                      x,y,z  =     1_555 Check
PLAT432_ALERT_2_G Short Inter X...Y Contact  Br1      ..C1              3.22 Ang.
                                                      x,y,z  =     1_555 Check
PLAT432_ALERT_2_G Short Inter X...Y Contact  Br2      ..C5              3.16 Ang.
```

```
                                                      x,y,z  =      1_555 Check
PLAT432_ALERT_2_G Short Inter X...Y Contact  Br2      ..C2               3.21 Ang.
                                                      x,y,z  =      1_555 Check
PLAT432_ALERT_2_G Short Inter X...Y Contact  Br2      ..C4               3.22 Ang.
                                                      x,y,z  =      1_555 Check
PLAT432_ALERT_2_G Short Inter X...Y Contact  Br3      ..C6               3.13 Ang.
                                                      x,y,z  =      1_555 Check
PLAT432_ALERT_2_G Short Inter X...Y Contact  Br3      ..C1               3.18 Ang.
                                                      x,y,z  =      1_555 Check
PLAT432_ALERT_2_G Short Inter X...Y Contact  Br3      ..C5               3.27 Ang.
                                                      x,y,z  =      1_555 Check
PLAT432_ALERT_2_G Short Inter X...Y Contact  O6       ..C7               2.98 Ang.
                                                    x,1+y,z  =      1_565 Check
PLAT432_ALERT_2_G Short Inter X...Y Contact  N4       ..C19              3.04 Ang.
                                                      x,y,z  =      1_555 Check
PLAT720_ALERT_4_G Number of Unusual/Non-Standard Labels ..........          2 Note
PLAT789_ALERT_4_G Atoms with Negative _atom_site_disorder_group  #         12 Check
PLAT790_ALERT_4_G Centre of Gravity not Within Unit Cell: Resd.  #          4 Note
              C4 H4 Br N O2
PLAT790_ALERT_4_G Centre of Gravity not Within Unit Cell: Resd.  #          6 Note
              C H2 Cl2
PLAT860_ALERT_3_G Number of Least-Squares Restraints .............         36 Note
PLAT883_ALERT_1_G No Info/Value for _atom_sites_solution_primary .     Please Do !
PLAT910_ALERT_3_G Missing # of FCF Reflection(s) Below Theta(Min).          1 Note
PLAT978_ALERT_2_G Number C-C Bonds with Positive Residual Density.          0 Info
```

---

   3 **ALERT level A** = Most likely a serious problem - resolve or explain
   0 **ALERT level B** = A potentially serious problem, consider carefully
   5 **ALERT level C** = Check. Ensure it is not caused by an omission or oversight
  38 **ALERT level G** = General information/check it is not something unexpected

```
   4 ALERT type 1 CIF construction/syntax error, inconsistent or missing data
  18 ALERT type 2 Indicator that the structure model may be wrong or deficient
   5 ALERT type 3 Indicator that the structure quality may be low
  19 ALERT type 4 Improvement, methodology, query or suggestion
   0 ALERT type 5 Informative message, check
```

It is advisable to attempt to resolve as many as possible of the alerts in all categories. Often the minor alerts point to easily fixed oversights, errors and omissions in your CIF or refinement strategy, so attention to these fine details can be worthwhile. In order to resolve some of the more serious problems it may be necessary to carry out additional measurements or structure refinements. However, the purpose of your study may justify the reported deviations and the more serious of these should normally be commented upon in the discussion or experimental section of a paper or in the "special_details" fields of the CIF. checkCIF was carefully designed to identify outliers and unusual parameters, but every test has its limitations and alerts that are not important in a particular case may appear. Conversely, the absence of alerts does not guarantee there are no aspects of the results needing attention. It is up to the individual to critically assess their own results and, if necessary, seek expert advice.

Publication of your CIF in IUCr journals

A basic structural check has been run on your CIF. These basic checks will be run on all CIFs submitted for publication in IUCr journals (*Acta Crystallographica*, *Journal of Applied Crystallography*, *Journal of Synchrotron Radiation*); however, if you intend to submit to *Acta Crystallographica Section C* or *E* or *IUCrData*, you should make sure that full publication checks are run on the final version of your CIF prior to submission.

Publication of your CIF in other journals

Please refer to the *Notes for Authors* of the relevant journal for any special instructions relating to CIF submission.

PLATON version of 18/09/2020; check.def file version of 20/08/2020

Datablock HMTA-NBS_4 - ellipsoid plot

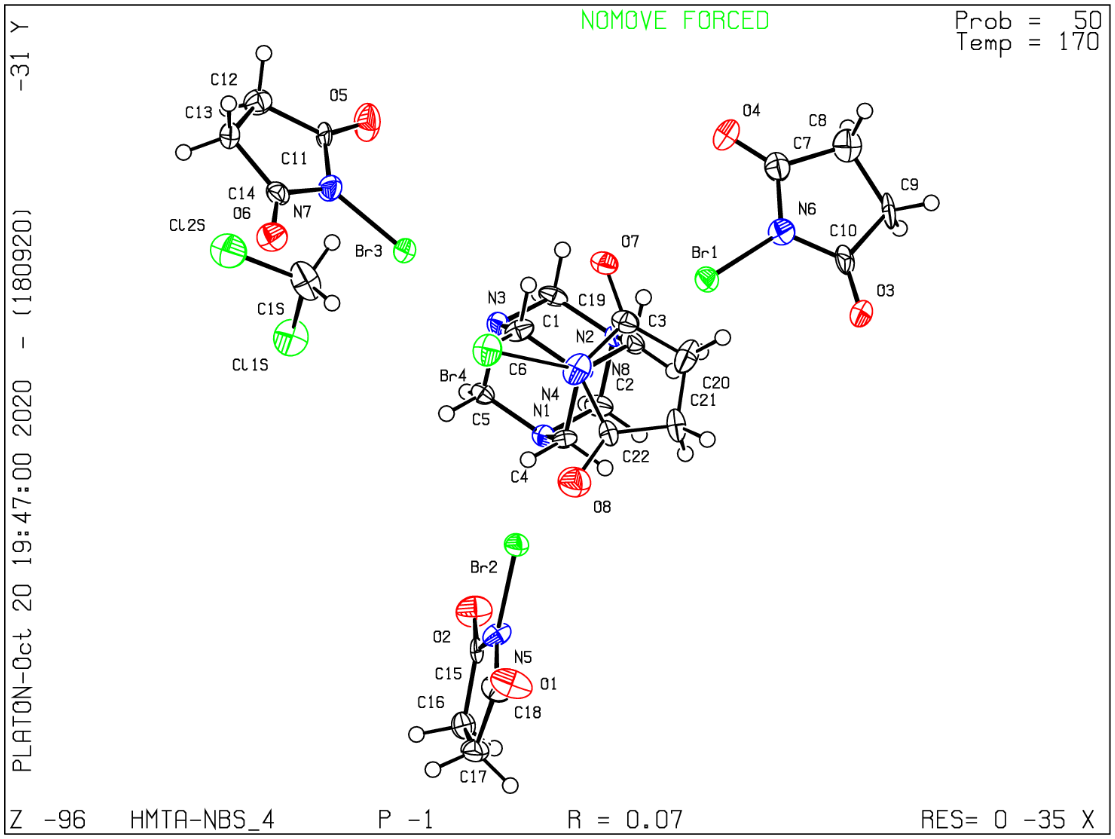

Supplement: Supplementary file 2 [file Data_Sheet_2.ZIP › CIF_MS 623595/[HMTA][NBS]4_checkcif.pdf]
