# Supplementary material for: Short X···N Halogen Bonds With Hexamethylenetetraamine as the Acceptor
Source: Front Chem. 2021 Apr 29;9:623595. doi: 10.3389/fchem.2021.623595 (PMC8116742; doi:10.3389/fchem.2021.623595)

checkCIF/PLATON report

Structure factors have been supplied for datablock(s) HMTA_Br-Cl_2

THIS REPORT IS FOR GUIDANCE ONLY. IF USED AS PART OF A REVIEW PROCEDURE FOR PUBLICATION, IT SHOULD NOT REPLACE THE EXPERTISE OF AN EXPERIENCED CRYSTALLOGRAPHIC REFEREE.

No syntax errors found. CIF dictionary Interpreting this report

Datablock: HMTA_Br-Cl_2

```
Bond precision:  N- C = 0.0051 A         Wavelength=0.71073

Cell:            a=6.0091(12)     b=14.422(3)       c=13.655(3)
                 alpha=90         beta=92.17(3)     gamma=90
Temperature:     170 K

                Calculated                 Reported
Volume          1182.5(4)                  1182.5(4)
Space group     P 21/c                     P 1 21/c 1
Hall group      -P 2ybc                    -P 2ybc
Moiety formula  C6 H12 Br2 Cl2 N4          C6 H12 Br2 Cl2 N4
Sum formula     C6 H12 Br2 Cl2 N4          C6 H12 Br2 Cl2 N4
Mr              370.90                     370.92
Dx,g cm-3       2.083                      2.083
Z               4                          4
Mu (mm-1)       7.276                      7.277
F000            720.0                      720.0
F000'           719.19
h,k,lmax        7,17,16                    7,17,16
Nref            2143                       2144
Tmin,Tmax       0.257,0.388                0.579,0.746
Tmin'           0.194

Correction method= # Reported T Limits: Tmin=0.579 Tmax=0.746
AbsCorr = MULTI-SCAN

Data completeness= 1.000          Theta(max)= 25.248

R(reflections)= 0.0296( 1790)     wR2(reflections)= 0.0694( 2144)

S = 1.036                  Npar= 127
```

The following ALERTS were generated. Each ALERT has the format
test-name_ALERT_alert-type_alert-level.
Click on the hyperlinks for more details of the test.

Alert level B

PLAT230_ALERT_2_B Hirshfeld Test Diff for Br1 --Cl1 . 10.2 s.u.

Author Response: Due to unresolved disorder

PLAT230_ALERT_2_B Hirshfeld Test Diff for Br2 --Cl2 . 8.3 s.u.

Author Response: Due to unresolved disorder

Alert level G

PLAT012_ALERT_1_G No _shelx_res_checksum Found in CIF ...... Please Check
PLAT909_ALERT_3_G Percentage of I>2sig(I) Data at Theta(Max) Still 71% Note

---

0 **ALERT level A** = Most likely a serious problem - resolve or explain
2 **ALERT level B** = A potentially serious problem, consider carefully
0 **ALERT level C** = Check. Ensure it is not caused by an omission or oversight
2 **ALERT level G** = General information/check it is not something unexpected

1 ALERT type 1 CIF construction/syntax error, inconsistent or missing data
2 ALERT type 2 Indicator that the structure model may be wrong or deficient
1 ALERT type 3 Indicator that the structure quality may be low
0 ALERT type 4 Improvement, methodology, query or suggestion
0 ALERT type 5 Informative message, check

It is advisable to attempt to resolve as many as possible of the alerts in all categories. Often the minor alerts point to easily fixed oversights, errors and omissions in your CIF or refinement strategy, so attention to these fine details can be worthwhile. In order to resolve some of the more serious problems it may be necessary to carry out additional measurements or structure refinements. However, the purpose of your study may justify the reported deviations and the more serious of these should normally be commented upon in the discussion or experimental section of a paper or in the "special_details" fields of the CIF. checkCIF was carefully designed to identify outliers and unusual parameters, but every test has its limitations and alerts that are not important in a particular case may appear. Conversely, the absence of alerts does not guarantee there are no aspects of the results needing attention. It is up to the individual to critically assess their own results and, if necessary, seek expert advice.

Publication of your CIF in IUCr journals

A basic structural check has been run on your CIF. These basic checks will be run on all CIFs submitted for publication in IUCr journals (*Acta Crystallographica*, *Journal of Applied Crystallography*, *Journal of Synchrotron Radiation*); however, if you intend to submit to *Acta Crystallographica Section C* or *E* or *IUCrData*, you should make sure that full publication checks are run on the final version of your CIF prior to submission.

Publication of your CIF in other journals

Please refer to the *Notes for Authors* of the relevant journal for any special instructions relating to CIF submission.

PLATON version of 18/09/2020; check.def file version of 20/08/2020

Datablock HMTA_Br-Cl_2 - ellipsoid plot

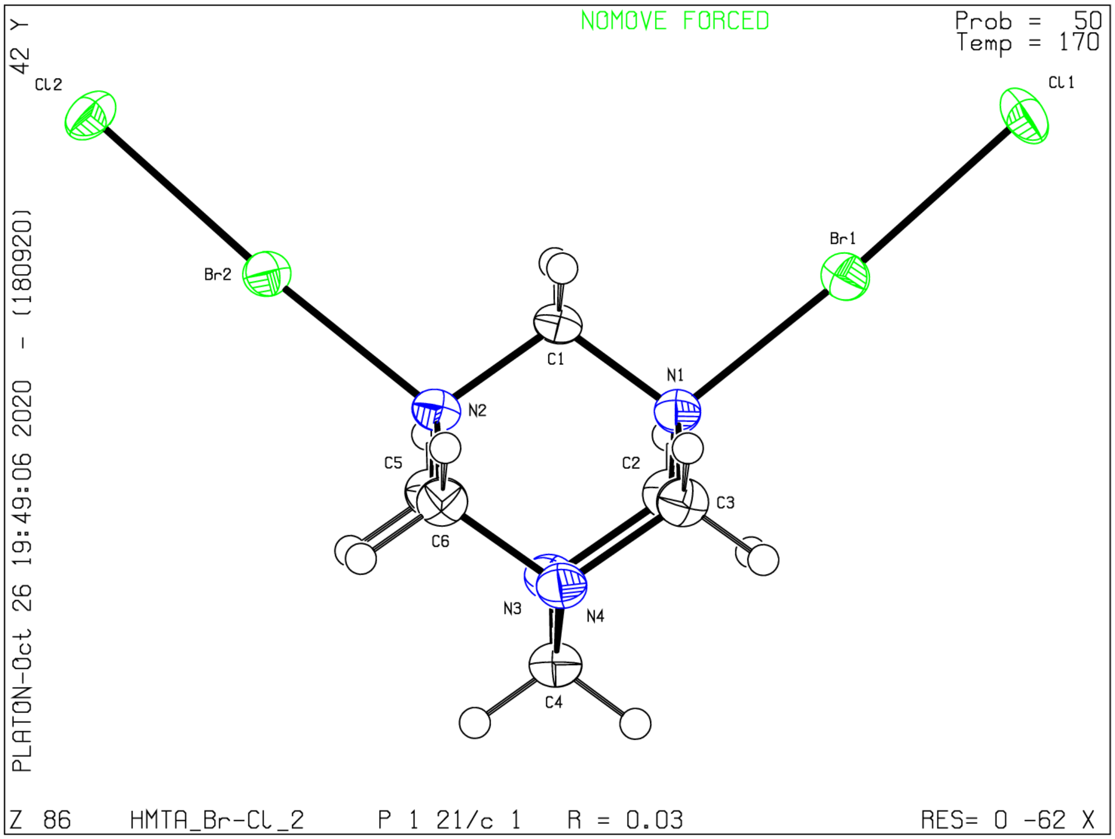

Supplement: Supplementary file 2 [file [HMTA][Br-Cl]2_checkcif.pdf › CIF_MS 623595/[HMTA][Br-Cl]2_checkcif.pdf]
