# Supplementary material for: Short X···N Halogen Bonds With Hexamethylenetetraamine as the Acceptor
Source: Front Chem. 2021 Apr 29;9:623595. doi: 10.3389/fchem.2021.623595 (PMC8116742; doi:10.3389/fchem.2021.623595)

checkCIF/PLATON report

Structure factors have been supplied for datablock(s) HMTA_I-Cl

THIS REPORT IS FOR GUIDANCE ONLY. IF USED AS PART OF A REVIEW PROCEDURE FOR PUBLICATION, IT SHOULD NOT REPLACE THE EXPERTISE OF AN EXPERIENCED CRYSTALLOGRAPHIC REFEREE.

No syntax errors found.   CIF dictionary   Interpreting this report

Datablock: HMTA_I-Cl

```
Bond precision:  N- C = 0.0081 A        Wavelength=0.71073

Cell:            a=5.9606(12)     b=13.646(3)      c=11.989(2)
                 alpha=90         beta=98.56(3)    gamma=90
Temperature:     170 K

                Calculated                 Reported
Volume          964.3(3)                   964.3(3)
Space group     P 21/c                     P 1 21/c 1
Hall group      -P 2ybc                    -P 2ybc
Moiety formula  C6 H12 Cl I N4             C6 H12 Cl I N4
Sum formula     C6 H12 Cl I N4             C6 H12 Cl I N4
Mr              302.55                     302.55
Dx,g cm-3       2.084                      2.084
Z               4                          4
Mu (mm-1)       3.552                      3.552
F000            584.0                      584.0
F000'           582.82
h,k,lmax        7,16,14                    7,16,14
Nref            1745                       1743
Tmin,Tmax       0.632,0.780                0.521,0.746
Tmin'           0.602

Correction method= # Reported T Limits: Tmin=0.521 Tmax=0.746
AbsCorr = MULTI-SCAN

Data completeness= 0.999          Theta(max)= 25.249

R(reflections)= 0.0378( 1474)     wR2(reflections)= 0.0819( 1743)

S = 1.066                 Npar= 109
```

The following ALERTS were generated. Each ALERT has the format
test-name_ALERT_alert-type_alert-level.
Click on the hyperlinks for more details of the test.

Alert level B

PLAT230_ALERT_2_B Hirshfeld Test Diff for I1 --Cl1 . 9.5 s.u.

Author Response: Due to unresolved disorder

Alert level C

PLAT906_ALERT_3_C Large K Value in the Analysis of Variance ...... 2.771 Check
PLAT911_ALERT_3_C Missing FCF Refl Between Thmin & STh/L= 0.600 2 Report

---

Alert level G

PLAT012_ALERT_1_G No _shelx_res_checksum Found in CIF ...... Please Check
PLAT083_ALERT_2_G SHELXL Second Parameter in WGHT Unusually Large 5.25 Why ?
PLAT909_ALERT_3_G Percentage of I>2sig(I) Data at Theta(Max) Still 75% Note
PLAT913_ALERT_3_G Missing # of Very Strong Reflections in FCF .... 1 Note
PLAT933_ALERT_2_G Number of OMIT Records in Embedded .res File ... 1 Note

---

0 **ALERT level A** = Most likely a serious problem - resolve or explain
1 **ALERT level B** = A potentially serious problem, consider carefully
2 **ALERT level C** = Check. Ensure it is not caused by an omission or oversight
5 **ALERT level G** = General information/check it is not something unexpected

1 ALERT type 1 CIF construction/syntax error, inconsistent or missing data
3 ALERT type 2 Indicator that the structure model may be wrong or deficient
4 ALERT type 3 Indicator that the structure quality may be low
0 ALERT type 4 Improvement, methodology, query or suggestion
0 ALERT type 5 Informative message, check

It is advisable to attempt to resolve as many as possible of the alerts in all categories. Often the minor alerts point to easily fixed oversights, errors and omissions in your CIF or refinement strategy, so attention to these fine details can be worthwhile. In order to resolve some of the more serious problems it may be necessary to carry out additional measurements or structure refinements. However, the purpose of your study may justify the reported deviations and the more serious of these should normally be commented upon in the discussion or experimental section of a paper or in the "special_details" fields of the CIF. checkCIF was carefully designed to identify outliers and unusual parameters, but every test has its limitations and alerts that are not important in a particular case may appear. Conversely, the absence of alerts does not guarantee there are no aspects of the results needing attention. It is up to the individual to critically assess their own results and, if necessary, seek expert advice.

Publication of your CIF in IUCr journals

A basic structural check has been run on your CIF. These basic checks will be run on all CIFs submitted for publication in IUCr journals (*Acta Crystallographica*, *Journal of Applied Crystallography*, *Journal of Synchrotron Radiation*); however, if you intend to submit to *Acta Crystallographica Section C* or *E* or *IUCrData*, you should make sure that full publication checks are run on the final version of your CIF prior to submission.

Publication of your CIF in other journals

Please refer to the *Notes for Authors* of the relevant journal for any special instructions relating to CIF submission.

PLATON version of 18/09/2020; check.def file version of 20/08/2020

Datablock HMTA_I-Cl - ellipsoid plot

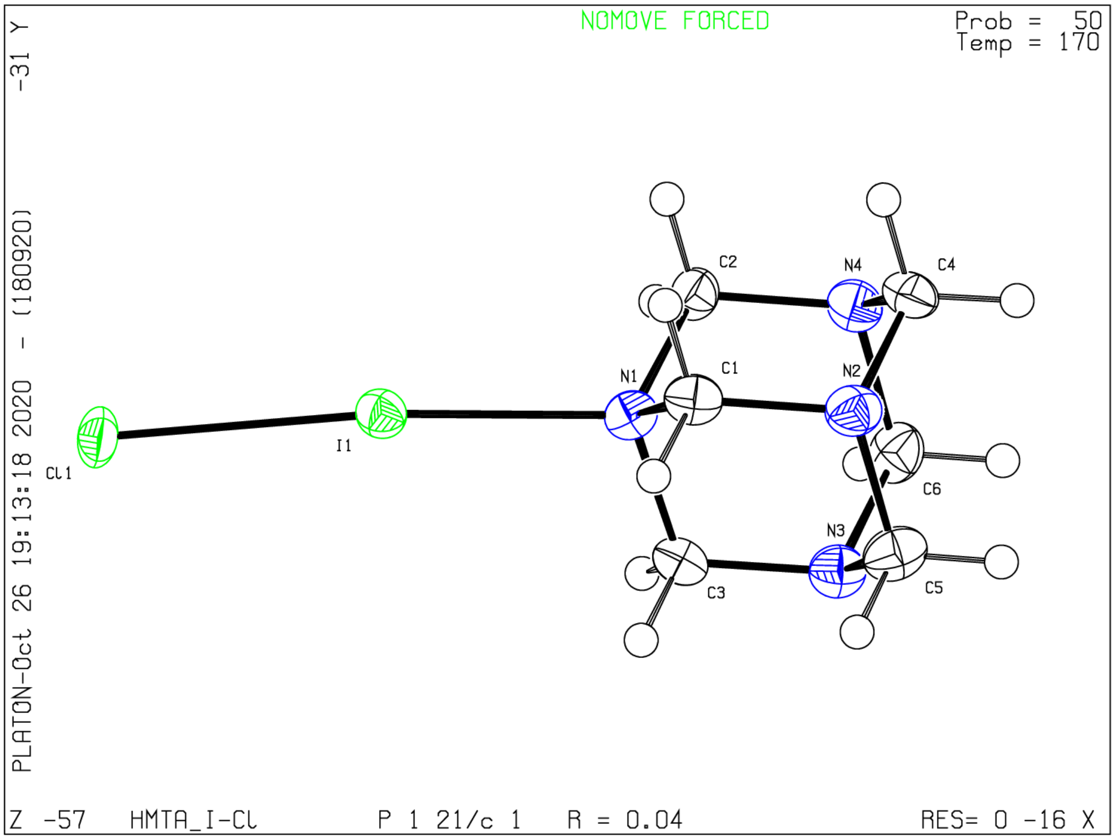

Supplement: Supplementary file 2 [file [HMTA][I-Cl]_checkcifpdf.pdf › CIF_MS 623595/[HMTA][I-Cl]_checkcifpdf.pdf]
