# Supplementary material for: Short X···N Halogen Bonds With Hexamethylenetetraamine as the Acceptor
Source: Front Chem. 2021 Apr 29;9:623595. doi: 10.3389/fchem.2021.623595 (PMC8116742; doi:10.3389/fchem.2021.623595)

checkCIF/PLATON report

Structure factors have been supplied for datablock(s) HMTA_I-I

THIS REPORT IS FOR GUIDANCE ONLY. IF USED AS PART OF A REVIEW PROCEDURE FOR PUBLICATION, IT SHOULD NOT REPLACE THE EXPERTISE OF AN EXPERIENCED CRYSTALLOGRAPHIC REFEREE.

No syntax errors found.   CIF dictionary   Interpreting this report

Datablock: HMTA_I-I

```
Bond precision:  N- C = 0.0080 A         Wavelength=0.71073

Cell:             a=9.2388(18)      b=7.6121(15)      c=15.423(3)
                  alpha=90          beta=90           gamma=90
Temperature:      170 K

                Calculated                  Reported
Volume          1084.7(4)                   1084.6(4)
Space group     C m c 21                    C m c 21
Hall group      C 2c -2                     C 2c -2
Moiety formula  C6 H12 I2 N4                C6 H12 I2 N4
Sum formula     C6 H12 I2 N4                C6 H12 I2 N4
Mr              394.00                      394.00
Dx,g cm-3       2.413                       2.413
Z               4                           4
Mu (mm-1)       5.758                       5.759
F000            728.0                       728.0
F000'           724.43
h,k,lmax        12,10,20                    12,10,20
Nref            1514[ 785]                  1505
Tmin,Tmax       0.348,0.562                 0.437,0.746
Tmin'           0.322

Correction method= # Reported T Limits: Tmin=0.437 Tmax=0.746
AbsCorr = MULTI-SCAN

Data completeness= 1.92/0.99      Theta(max)= 28.870

R(reflections)= 0.0160( 1483)     wR2(reflections)= 0.0369( 1505)

S = 1.097                  Npar= 64
```

The following ALERTS were generated. Each ALERT has the format
test-name_ALERT_alert-type_alert-level.
Click on the hyperlinks for more details of the test.

Alert level B

```
PLAT230_ALERT_2_B Hirshfeld Test Diff for    I1       --I2       .        9.0 s.u.
```

Author Response: Due to unresolved disorder

Alert level C

```
STRVA01_ALERT_4_C          Flack test results are ambiguous.
           From the CIF: _refine_ls_abs_structure_Flack    0.350
           From the CIF: _refine_ls_abs_structure_Flack_su    0.014
PLAT927_ALERT_1_C Reported and Calculated  wR2 Differ by .........     0.0014 Check
PLAT987_ALERT_1_C The Flack x is >> 0 -  Do a BASF/TWIN Refinement     Please Check
```

Alert level G

```
PLAT012_ALERT_1_G No       _shelx_res_checksum Found in CIF ......     Please Check
PLAT300_ALERT_4_G Atom Site Occupancy of H4A       Constrained at        0.5 Check
PLAT300_ALERT_4_G Atom Site Occupancy of H4B       Constrained at        0.5 Check
PLAT883_ALERT_1_G No Info/Value for _atom_sites_solution_primary .     Please Do !
PLAT912_ALERT_4_G Missing # of FCF Reflections Above STh/L=  0.600          3 Note
PLAT913_ALERT_3_G Missing # of Very Strong Reflections in FCF ....          1 Note
```

---

0 **ALERT level A** = Most likely a serious problem - resolve or explain
1 **ALERT level B** = A potentially serious problem, consider carefully
3 **ALERT level C** = Check. Ensure it is not caused by an omission or oversight
6 **ALERT level G** = General information/check it is not something unexpected

4 ALERT type 1 CIF construction/syntax error, inconsistent or missing data
1 ALERT type 2 Indicator that the structure model may be wrong or deficient
1 ALERT type 3 Indicator that the structure quality may be low
4 ALERT type 4 Improvement, methodology, query or suggestion
0 ALERT type 5 Informative message, check

It is advisable to attempt to resolve as many as possible of the alerts in all categories. Often the minor alerts point to easily fixed oversights, errors and omissions in your CIF or refinement strategy, so attention to these fine details can be worthwhile. In order to resolve some of the more serious problems it may be necessary to carry out additional measurements or structure refinements. However, the purpose of your study may justify the reported deviations and the more serious of these should normally be commented upon in the discussion or experimental section of a paper or in the "special_details" fields of the CIF. checkCIF was carefully designed to identify outliers and unusual parameters, but every test has its limitations and alerts that are not important in a particular case may appear. Conversely, the absence of alerts does not guarantee there are no aspects of the results needing attention. It is up to the individual to critically assess their own results and, if necessary, seek expert advice.

Publication of your CIF in IUCr journals

A basic structural check has been run on your CIF. These basic checks will be run on all CIFs submitted for publication in IUCr journals (*Acta Crystallographica*, *Journal of Applied Crystallography*, *Journal of Synchrotron Radiation*); however, if you intend to submit to *Acta Crystallographica Section C* or *E* or *IUCrData*, you should make sure that full publication checks are run on the final version of your CIF prior to submission.

Publication of your CIF in other journals

Please refer to the *Notes for Authors* of the relevant journal for any special instructions relating to CIF submission.

PLATON version of 18/09/2020; check.def file version of 20/08/2020

Datablock HMTA_I-I - ellipsoid plot

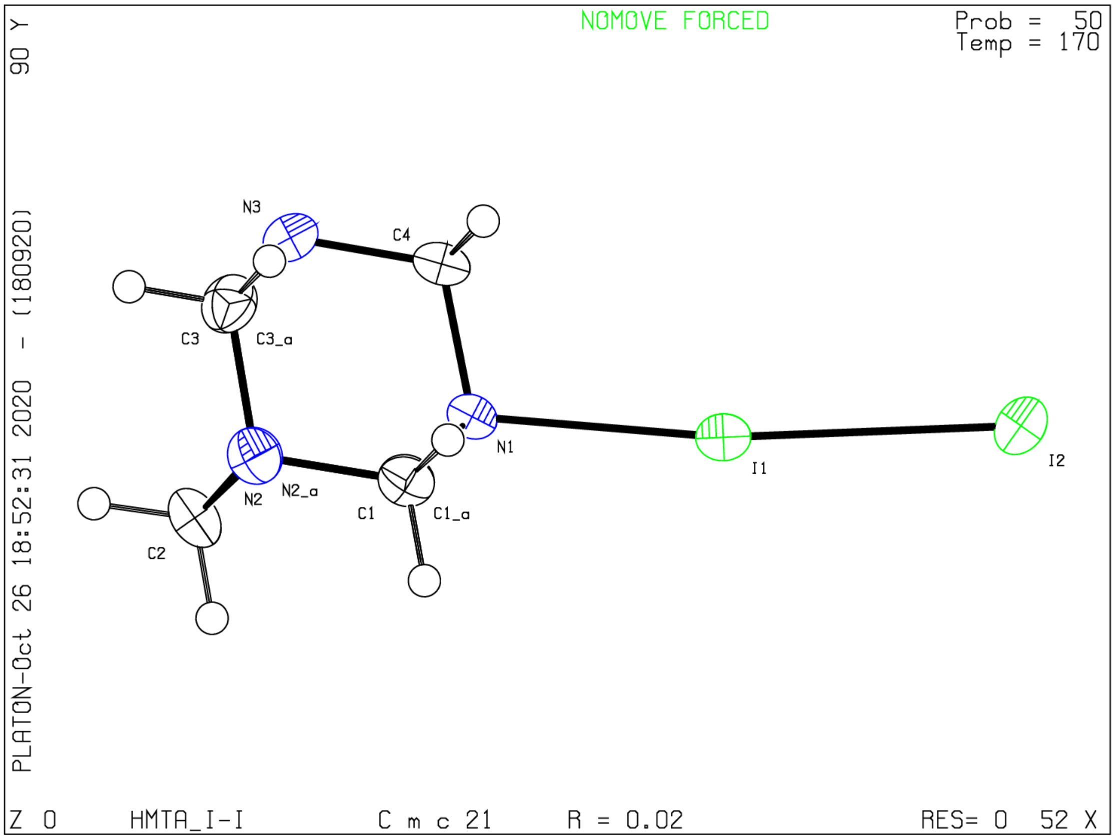

Supplement: Supplementary file 2 [file [HMTA][I-I]_checkcif.pdf › CIF_MS 623595/[HMTA][I-I]_checkcif.pdf]
