# Supplementary material for: Short X···N Halogen Bonds With Hexamethylenetetraamine as the Acceptor
Source: Front Chem. 2021 Apr 29;9:623595. doi: 10.3389/fchem.2021.623595 (PMC8116742; doi:10.3389/fchem.2021.623595)

checkCIF/PLATON report

Structure factors have been supplied for datablock(s) HMTA-NBP_2

THIS REPORT IS FOR GUIDANCE ONLY. IF USED AS PART OF A REVIEW PROCEDURE FOR PUBLICATION, IT SHOULD NOT REPLACE THE EXPERTISE OF AN EXPERIENCED CRYSTALLOGRAPHIC REFEREE.

No syntax errors found.   CIF dictionary   Interpreting this report

Datablock: HMTA-NBP_2

Bond precision: C-C = 0.0126 A   Wavelength=0.71073

Cell:	a=31.646(6)	b=5.7998(7)	c=5.9963(9)
	alpha=90	beta=90	gamma=90
Temperature:	120 K		

	Calculated	Reported
Volume	1100.6(3)	1100.5(3)
Space group	P m n 21	P m n 21
Hall group	P 2ac -2	P 2ac -2
Moiety formula	C8 H4 Br N O2, 0.5(C6 H12 N4)	C8 H4 Br N O2, C3 H6 N2
Sum formula	C11 H10 Br N3 O2	C11 H10 Br N3 O2
Mr	296.12	296.13
Dx,g cm-3	1.787	1.787
Z	4	4
Mu (mm-1)	3.728	3.728
F000	592.0	592.0
F000’	591.09	
h,k,lmax	45,8,8	44,8,8
Nref	3397[1848]	3206
Tmin,Tmax	0.778,0.782	0.817,1.000
Tmin’	0.763	

Correction method= # Reported T Limits: Tmin=0.817 Tmax=1.000
AbsCorr = GAUSSIAN

Data completeness= 1.73/0.94   Theta(max)= 30.455

R(reflections)= 0.0700(2137)   wR2(reflections)= 0.0874(3206)

S = 1.010   Npar= 156

The following ALERTS were generated. Each ALERT has the format
test-name_ALERT_alert-type_alert-level.
Click on the hyperlinks for more details of the test.

Alert level A

```
PLAT431_ALERT_2_A Short Inter HL..A Contact  Br1      ..N1       .       2.38 Ang.
                                                      x,y,z  =      1_555 Check
```

Author Response: Halogen bonding

Alert level C

```
RINTA01_ALERT_3_C  The value of Rint is greater than 0.12
            Rint given   0.143
PLAT213_ALERT_2_C Atom C6              has ADP max/min Ratio .....        3.7 prolat
PLAT341_ALERT_3_C Low Bond Precision on  C-C Bonds ...............    0.01262 Ang.
PLAT790_ALERT_4_C Centre of Gravity not Within Unit Cell: Resd.  #          1 Note
              C8 H4 Br N O2
PLAT906_ALERT_3_C Large K Value in the Analysis of Variance ......      6.086 Check
PLAT906_ALERT_3_C Large K Value in the Analysis of Variance ......      2.382 Check
```

Alert level G

```
PLAT012_ALERT_1_G No       _shelx_res_checksum Found in CIF ......     Please Check
PLAT020_ALERT_3_G The Value of Rint is Greater Than 0.12 .........      0.143 Report
PLAT042_ALERT_1_G Calc. and Reported MoietyFormula Strings  Differ     Please Check
PLAT171_ALERT_4_G The CIF-Embedded .res File Contains EADP Records          1 Report
PLAT300_ALERT_4_G Atom Site Occupancy of H2A        Constrained at        0.5 Check
PLAT300_ALERT_4_G Atom Site Occupancy of H2B        Constrained at        0.5 Check
PLAT432_ALERT_2_G Short Inter X...Y Contact  Br1      ..C4               3.09 Ang.
                                                      x,y,z  =      1_555 Check
PLAT432_ALERT_2_G Short Inter X...Y Contact  Br1      ..C1               3.21 Ang.
                                                      x,y,z  =      1_555 Check
PLAT883_ALERT_1_G No Info/Value for _atom_sites_solution_primary .     Please Do !
PLAT910_ALERT_3_G Missing # of FCF Reflection(s) Below Theta(Min).          2 Note
PLAT912_ALERT_4_G Missing # of FCF Reflections Above STh/L=  0.600         53 Note
PLAT963_ALERT_2_G Both SHELXL WEIGHT Parameter Values Zero .......     Please Check
PLAT978_ALERT_2_G Number C-C Bonds with Positive Residual Density.          0 Info
```

---

1 **ALERT level A** = Most likely a serious problem - resolve or explain
0 **ALERT level B** = A potentially serious problem, consider carefully
6 **ALERT level C** = Check. Ensure it is not caused by an omission or oversight
13 **ALERT level G** = General information/check it is not something unexpected

3 ALERT type 1 CIF construction/syntax error, inconsistent or missing data
6 ALERT type 2 Indicator that the structure model may be wrong or deficient
6 ALERT type 3 Indicator that the structure quality may be low
5 ALERT type 4 Improvement, methodology, query or suggestion
0 ALERT type 5 Informative message, check

It is advisable to attempt to resolve as many as possible of the alerts in all categories. Often the minor alerts point to easily fixed oversights, errors and omissions in your CIF or refinement strategy, so attention to these fine details can be worthwhile. In order to resolve some of the more serious problems it may be necessary to carry out additional measurements or structure refinements. However, the purpose of your study may justify the reported deviations and the more serious of these should normally be commented upon in the discussion or experimental section of a paper or in the "special_details" fields of the CIF. checkCIF was carefully designed to identify outliers and unusual parameters, but every test has its limitations and alerts that are not important in a particular case may appear. Conversely, the absence of alerts does not guarantee there are no aspects of the results needing attention. It is up to the individual to critically assess their own results and, if necessary, seek expert advice.

Publication of your CIF in IUCr journals

A basic structural check has been run on your CIF. These basic checks will be run on all CIFs submitted for publication in IUCr journals (*Acta Crystallographica*, *Journal of Applied Crystallography*, *Journal of Synchrotron Radiation*); however, if you intend to submit to *Acta Crystallographica Section C* or *E* or *IUCrData*, you should make sure that full publication checks are run on the final version of your CIF prior to submission.

Publication of your CIF in other journals

Please refer to the *Notes for Authors* of the relevant journal for any special instructions relating to CIF submission.

PLATON version of 18/09/2020; check.def file version of 20/08/2020

Datablock HMTA-NBP_2 - ellipsoid plot

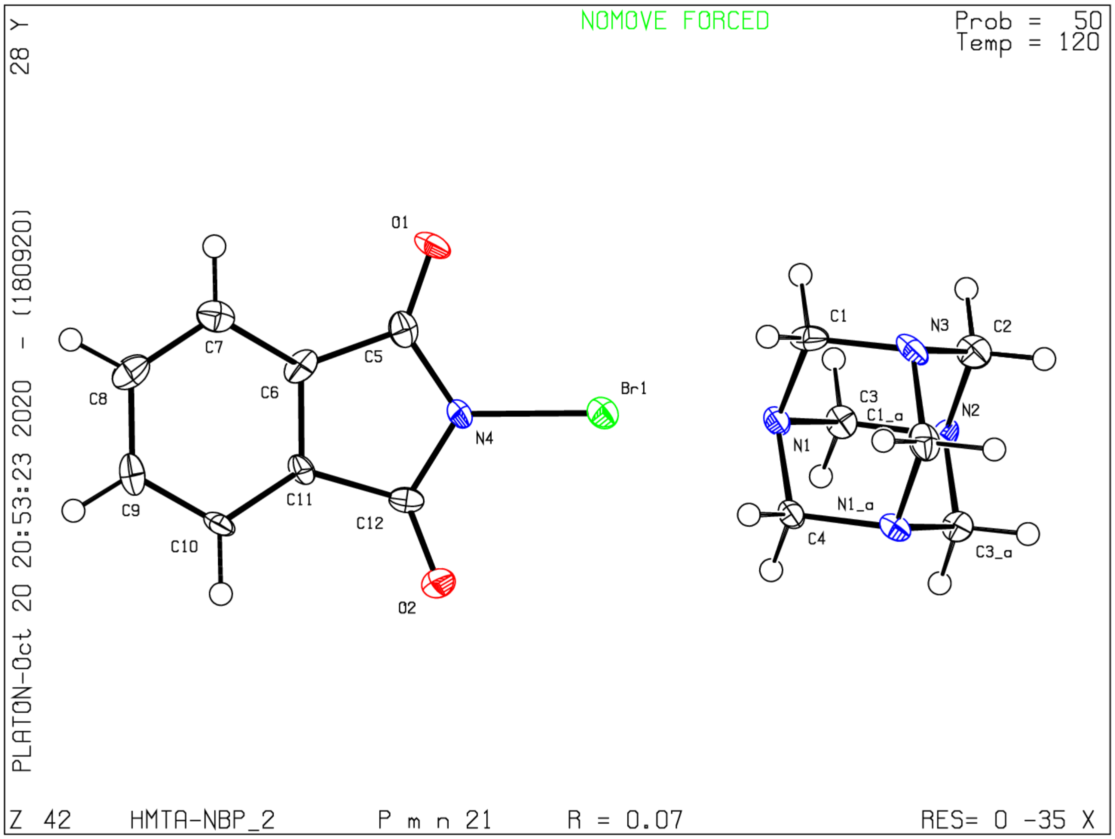

Supplement: Supplementary file 2 [file [HMTA][NBP]2_checkcif.pdf › CIF_MS 623595/[HMTA][NBP]2_checkcif.pdf]
