# Supplementary material for: Short X···N Halogen Bonds With Hexamethylenetetraamine as the Acceptor
Source: Front Chem. 2021 Apr 29;9:623595. doi: 10.3389/fchem.2021.623595 (PMC8116742; doi:10.3389/fchem.2021.623595)

checkCIF/PLATON report

Structure factors have been supplied for datablock(s) HMTA-NISac

THIS REPORT IS FOR GUIDANCE ONLY. IF USED AS PART OF A REVIEW PROCEDURE FOR PUBLICATION, IT SHOULD NOT REPLACE THE EXPERTISE OF AN EXPERIENCED CRYSTALLOGRAPHIC REFEREE.

No syntax errors found. CIF dictionary Interpreting this report

Datablock: HMTA-NISac

```
Bond precision:  C-C = 0.0357 A          Wavelength=0.71073

Cell:            a=7.7514(16)     b=7.6524(15)      c=13.862(3)
                 alpha=90         beta=97.87(3)     gamma=90
Temperature:     170 K

                Calculated                Reported
Volume          814.5(3)                  814.5(3)
Space group     P 21                      P 1 21 1
Hall group      P 2yb                     P 2yb
Moiety formula  C13 H16 I N5 O3 S         C13 H16 I N5 O3 S
Sum formula     C13 H16 I N5 O3 S         C13 H16 I N5 O3 S
Mr              449.27                    449.27
Dx,g cm-3       1.832                     1.832
Z               2                         2
Mu (mm-1)       2.116                     2.116
F000            444.0                     444.0
F000'           443.45
h,k,lmax        9,9,16                    9,9,16
Nref            2940[ 1591]               2811
Tmin,Tmax       0.796,0.900               0.614,0.746
Tmin'           0.760

Correction method= # Reported T Limits: Tmin=0.614 Tmax=0.746
AbsCorr = MULTI-SCAN

Data completeness= 1.77/0.96      Theta(max)= 25.249

R(reflections)= 0.0935( 1481)     wR2(reflections)= 0.1330( 2811)

S = 1.131                  Npar= 196
```

The following ALERTS were generated. Each ALERT has the format
test-name_ALERT_alert-type_alert-level.
Click on the hyperlinks for more details of the test.

Alert level B

```
PLAT342_ALERT_3_B Low Bond Precision on  C-C Bonds ...............    0.03571 Ang.
```

Author Response: Due to unresolved disorder

Alert level C

```
PLAT213_ALERT_2_C Atom N1              has ADP max/min Ratio .....        3.4 oblate
PLAT234_ALERT_4_C Large Hirshfeld Difference S1       --O3       .       0.18 Ang.
PLAT241_ALERT_2_C High   'MainMol' Ueq as Compared to Neighbors of        C10 Check
PLAT906_ALERT_3_C Large K Value in the Analysis of Variance ......      4.753 Check
PLAT971_ALERT_2_C Check Calcd Resid. Dens.  0.14A   From I1              1.56 eA-3
PLAT975_ALERT_2_C Check Calcd Resid. Dens.  0.92A   From N3              0.55 eA-3
PLAT976_ALERT_2_C Check Calcd Resid. Dens.  0.84A   From N1             -0.51 eA-3
PLAT976_ALERT_2_C Check Calcd Resid. Dens.  1.00A   From O2             -0.46 eA-3
PLAT976_ALERT_2_C Check Calcd Resid. Dens.  0.62A   From O1             -0.45 eA-3
PLAT987_ALERT_1_C The Flack x is >> 0 -  Do a BASF/TWIN Refinement     Please Check
```

Alert level G

```
PLAT003_ALERT_2_G Number of Uiso or Uij Restrained non-H Atoms ...          1 Report
PLAT012_ALERT_1_G No       _shelx_res_checksum Found in CIF ......     Please Check
PLAT033_ALERT_4_G Flack x Value Deviates > 3.0 * sigma from Zero .      0.270 Note
PLAT171_ALERT_4_G The CIF-Embedded .res File Contains EADP Records          2 Report
PLAT186_ALERT_4_G The CIF-Embedded .res File Contains ISOR Records          1 Report
PLAT860_ALERT_3_G Number of Least-Squares Restraints .............          7 Note
PLAT910_ALERT_3_G Missing # of FCF Reflection(s) Below Theta(Min).          1 Note
PLAT978_ALERT_2_G Number C-C Bonds with Positive Residual Density.          0 Info
```

---

0 **ALERT level A** = Most likely a serious problem - resolve or explain
1 **ALERT level B** = A potentially serious problem, consider carefully
10 **ALERT level C** = Check. Ensure it is not caused by an omission or oversight
8 **ALERT level G** = General information/check it is not something unexpected

2 ALERT type 1 CIF construction/syntax error, inconsistent or missing data
9 ALERT type 2 Indicator that the structure model may be wrong or deficient
4 ALERT type 3 Indicator that the structure quality may be low
4 ALERT type 4 Improvement, methodology, query or suggestion
0 ALERT type 5 Informative message, check

It is advisable to attempt to resolve as many as possible of the alerts in all categories. Often the minor alerts point to easily fixed oversights, errors and omissions in your CIF or refinement strategy, so attention to these fine details can be worthwhile. In order to resolve some of the more serious problems it may be necessary to carry out additional measurements or structure refinements. However, the purpose of your study may justify the reported deviations and the more serious of these should normally be commented upon in the discussion or experimental section of a paper or in the "special_details" fields of the CIF. checkCIF was carefully designed to identify outliers and unusual parameters, but every test has its limitations and alerts that are not important in a particular case may appear. Conversely, the absence of alerts does not guarantee there are no aspects of the results needing attention. It is up to the individual to critically assess their own results and, if necessary, seek expert advice.

Publication of your CIF in IUCr journals

A basic structural check has been run on your CIF. These basic checks will be run on all CIFs submitted for publication in IUCr journals (*Acta Crystallographica*, *Journal of Applied Crystallography*, *Journal of Synchrotron Radiation*); however, if you intend to submit to *Acta Crystallographica Section C* or *E* or *IUCrData*, you should make sure that full publication checks are run on the final version of your CIF prior to submission.

Publication of your CIF in other journals

Please refer to the *Notes for Authors* of the relevant journal for any special instructions relating to CIF submission.

PLATON version of 18/09/2020; check.def file version of 20/08/2020

Datablock HMTA-NISac - ellipsoid plot

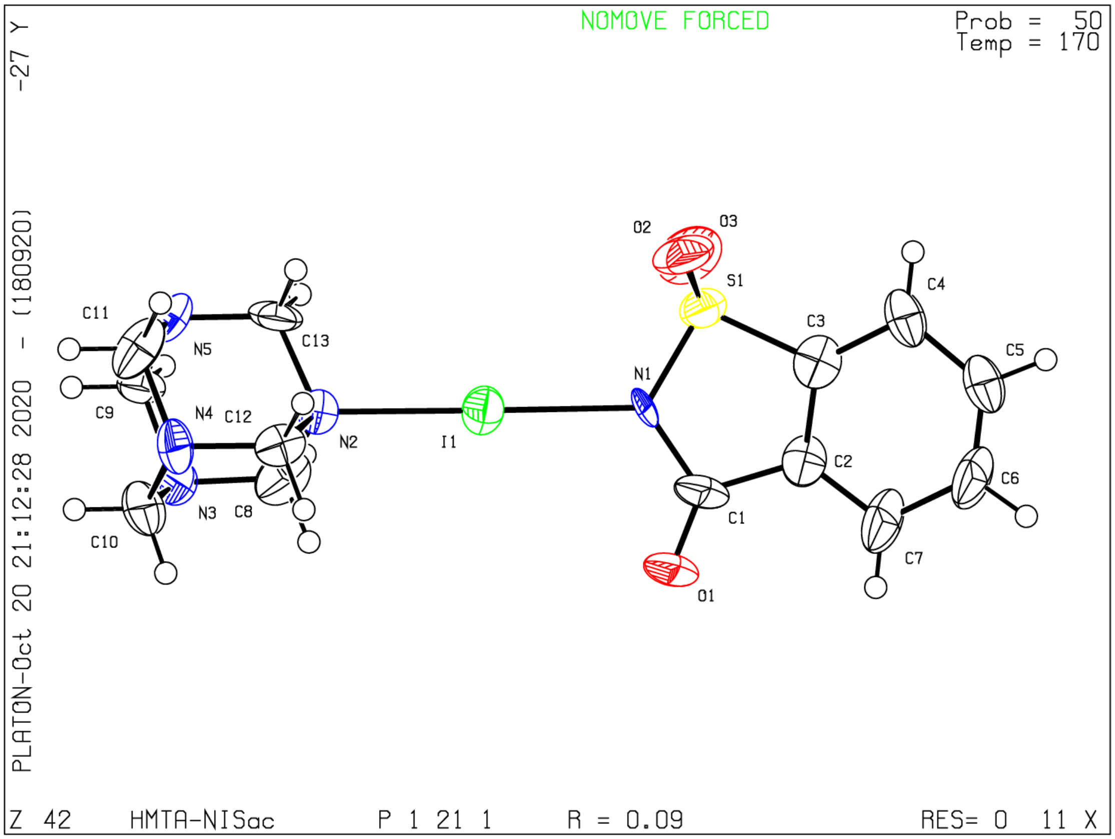

Supplement: Supplementary file 2 [file [HMTA][NISac]_checkcif.pdf › CIF_MS 623595/[HMTA][NISac]_checkcif.pdf]
